# Topical antibiotics as a major contextual hazard toward bacteremia within selective digestive decontamination studies: a meta-analysis

**DOI:** 10.1186/s12879-014-0714-x

**Published:** 2014-12-31

**Authors:** James C Hurley

**Affiliations:** Associate Professor, Rural Health Academic Center, Melbourne Medical School, University of Melbourne, Melbourne, Australia; Physician and Head of General Medicine, Internal Medicine Service, Ballarat Health Services, Ballarat, 3353 Australia; Chairman, Infection Control Committees, St John of God Hospital and Ballarat Health Services, Ballarat, VIC Australia

**Keywords:** Ventilator associated pneumonia, Bacteremia, Benchmarking, Antibiotic prophylaxis, Cross infection, Caterpillar plots

## Abstract

**Background:**

Among methods for preventing pneumonia and possibly also bacteremia in intensive care unit (ICU) patients, Selective Digestive Decontamination (SDD) appears most effective within randomized concurrent controlled trials (RCCT’s) although more recent trials have been cluster randomized. However, of the SDD components, whether protocolized parenteral antibiotic prophylaxis (PPAP) is required, and whether the topical antibiotic actually presents a contextual hazard, remain unresolved. The objective here is to compare the bacteremia rates and patterns of isolates in SDD-RCCT’s versus the broader evidence base.

**Methods:**

Bacteremia incidence proportion data were extracted from component (control and intervention) groups decanted from studies investigating antibiotic (SDD) or non-antibiotic methods of VAP prevention and summarized using random effects meta-analysis of study and group level data. A reference category of groups derived from purely observational studies without any prevention method under study provided a benchmark incidence.

**Results:**

Within SDD RCCTs, the mean bacteremia incidence among concurrent component groups not exposed to PPAP (27 control; 17.1%; 13.1-22.1% and 12 intervention groups; 16.2%; 9.1-27.3%) is double that of the benchmark bacteremia incidence derived from 39 benchmark groups (8.3; 6.8-10.2%) and also 20 control groups from studies of non-antibiotic methods (7.1%; 4.8 – 10.5). There is a selective increase in coagulase negative staphylococci (CNS) but not in *Pseudomonas aeruginosa* among bacteremia isolates within control groups of SDD-RCCT’s versus benchmark groups with data available.

**Conclusions:**

The topical antibiotic component of SDD presents a major contextual hazard toward bacteremia against which the PPAP component partially mitigates.

**Electronic supplementary material:**

The online version of this article (doi:10.1186/s12879-014-0714-x) contains supplementary material, which is available to authorized users.

## Background

Infections acquired by patients requiring a prolonged stay in the intensive care unit (ICU) have been studied extensively [1]-[112]. These are a leading cause of potentially preventable illness and death [113],[114]. In this patient group, the acquisition of colonizing bacteria is a key intermediary step toward the development of both bacteremia and ventilator associated pneumonia (VAP) [115],[116].

Among an extensive range of methods for the prevention of VAP in this patient group, Selective Digestive Decontamination (SDD) and Selective Oro-pharyngeal Decontamination (SOD) are of great interest for several reasons [117]-[121]. Firstly, as a counterfactual, the reduction in VAP incidence observed in 36 randomized concurrent controlled trials (RCCTs) of SDD is 65% [119] versus less than 40% for prevention methods that are non-antibiotic based. Second, SDD is postulated to have multi-site actions mediated against colonizing bacteria both at the oro-pharynx and gastro-intestinal tracts resulting in reductions in bacteremia as great as 27% [118],[120].

Thirdly, SDD is postulated to impart contextual effects mediated through cross colonization within the ICU. That SDD could influence the infection incidences beyond the intervention groups of concurrent design studies was postulated in the original 1984 SDD study [71] and others [60],[63] which as a consequence were either intentionally non-concurrent in design or more recently used cluster randomized design. Testing for the postulated SDD contextual effects on infection incidences is difficult due to the methodological and analytical challenges which cannot be adequately addressed within the confines of the typical single center RCCT.

Finally, SDD has been evaluated with at least five major variations in study design [121]; using either concurrent versus non-concurrent designs, with either or both of bacteremia and VAP as study end points, with different compositions of multi-component antibiotics in the SDD regimens, with SDD administered factorized as topical antibiotic prophylaxis alone or together with protocolized parenteral antibiotic prophylaxis (PPAP), with the PPAP component of SDD administered sometimes to the control groups in addition to the intervention groups (duplex studies), and with at risk ICU populations including varying proportions of trauma, medical and surgical patients under evaluation. This multiplicity of study designs creates a natural experiment in which the contextual effect of any group wide intervention, such as the factorized components of SDD might be inferred. This can be achieved through a calibration of bacteremia, or any other end point of interest, across component groups decanted from the various design types of these SDD studies versus component groups decanted from studies within the broader evidence base using methods analogous to those used in cluster randomized trials. In such a calibration, studies of non-antibiotic methods for the prevention of VAP are included to provide additional reference.

Such a calibration of the VAP end point among the component groups of 36 SDD studies with concurrent design reveals an SDD contextual hazard as follows; the VAP incidence is 14 percentage points higher among control groups [122] together with a selective increase in the proportion of *Staphylococci* [123] but not *Pseudomonas aeruginosa* [124] among the VAP isolates in both intervention and also control groups [123] of SDD-RCCTs versus observational study groups. Moreover these hazards are not seen among studies of non-antibiotic methods for the prevention of VAP.

## Methods

### Overview

The purpose of this analysis is to compare the bacteremia incidence and patterns of isolates in groups of ICU patients exposed directly or indirectly (contextually) to the topical and or parenteral components of SDD within RCCT’s versus component groups from studies within the broader evidence base that relates to the ICU patient group at risk of bacteremia and VAP. Of interest are comparisons of bacteremia not only versus other study designs of SDD but also versus studies of other interventions used to prevent infection in ICU patients in which the bacteremia incidence has been measured. Of secondary interest are comparisons of the VAP end point among these studies and the effect sizes of the various interventions that were under study against the two end points.

### Study selection

**Figure 1:**
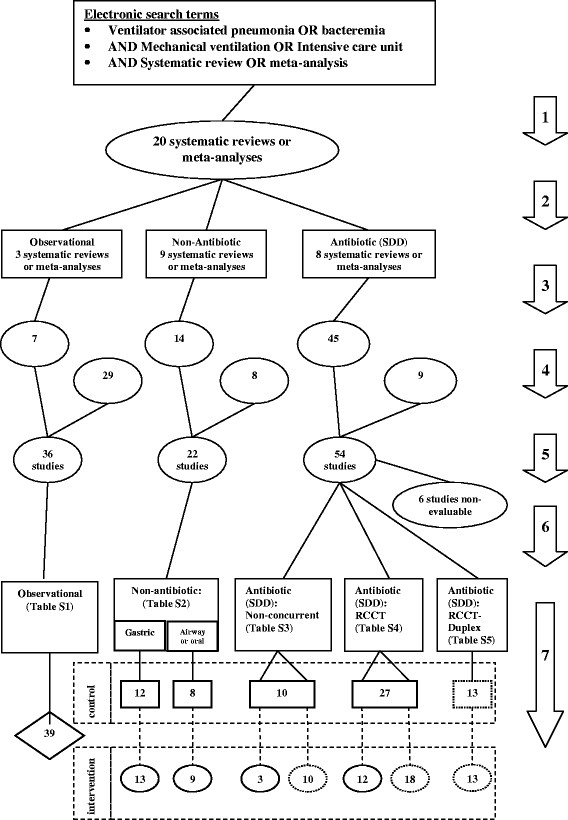
**Search method (numbered arrow 1) and streaming (arrow 2) of systematic reviews, screening (arrow 3, 4 & 5) and classification (arrow 6) of eligible studies, and decant and analytic plan (arrow 7) of component groups being control (rectangles) and intervention (ovals) groups from studies of VAP prevention methods and a reference category of observation (diamond) groups from cohorts of ICU patients without a pneumonia prevention method under study.** Dotted rectangles and ovals represent component groups within studies of antibiotic based methods of VAP prevention (SDD) which received protocolized parenteral antibiotic prophylaxis. Analytic plan; the vertical dotted lines connecting the component groups represent the group contrasts used towards the calculation of the counterfactual effects and the horizontal dotted rectangles represent the calibrations used toward the estimation of contextual effects among the component groups referent to the observation groups. Note; the total numbers do not tally as some systematic reviews provided studies in more than one category and some studies provided groups in more than one category.

**Figure 2:**
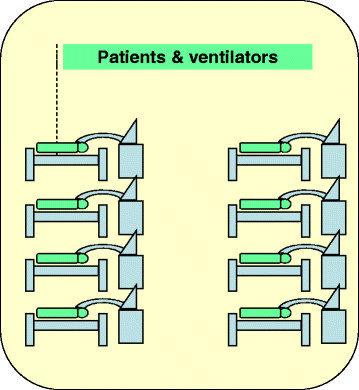
**Schematic of a group of patients receiving mechanical ventilation in an ICU without a VAP prevention method under study.**

**Figure 3:**
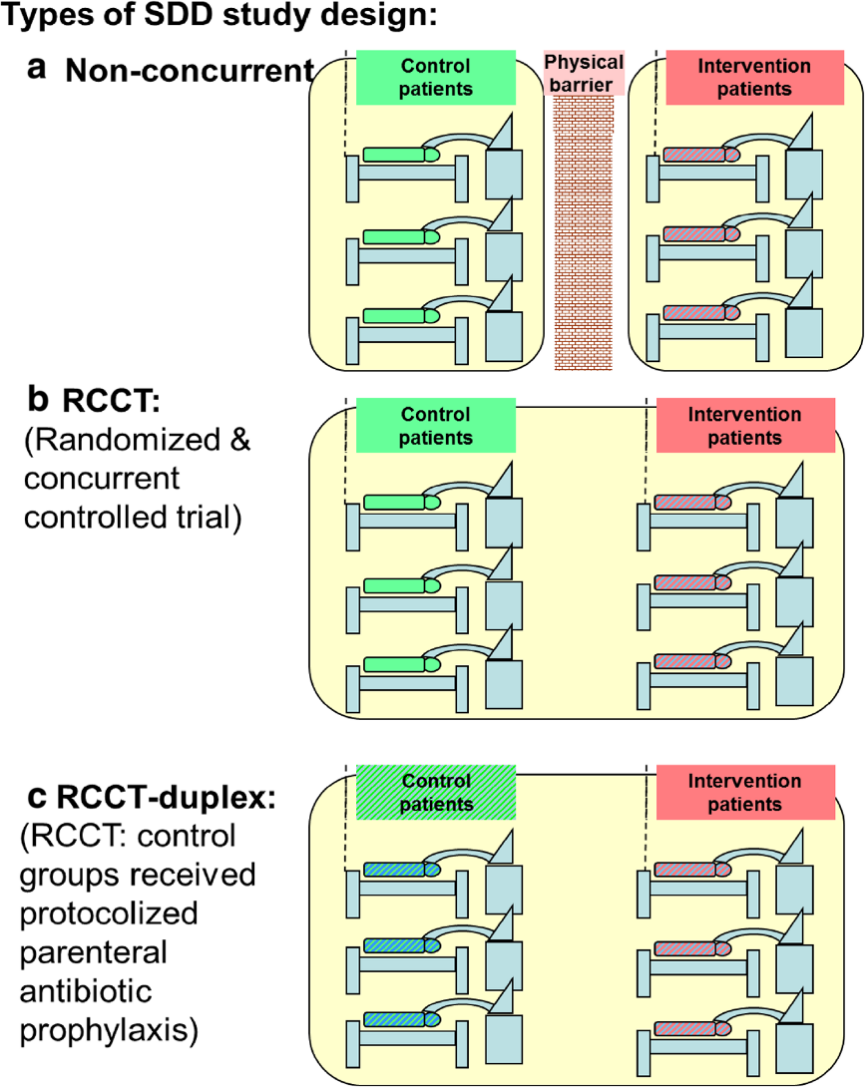
**Schematics of SDD study designs with intervention and control groups being either non-concurrent (a) or concurrent (b & c) and with intervention groups receiving prophylaxis with either or both protocolized parenteral and topical antibiotics (dual colour stripes; a, b & c) and with control group patients receiving the protocolized parenteral antibiotic prophylaxis component alone (RCCT-duplex studies; dual colour stripes; c); or not (monochrome; a & b).** Note the non-concurrent control and intervention patient groups were separated by a physical or temporal barrier (a).

The seven steps in the selection of studies and subsequent decanting of component groups and the plan of analysis is as depicted in Figure 1. These steps are detailed as follows;

1. An electronic search of PubMed, The Cochrane database and Google Scholar for systematic reviews containing potentially eligible studies was undertaken using the following search terms; “ventilator associated pneumonia”, “mechanical ventilation”, “intensive care unit”, “blood stream infection”, “bacteremia”, “meta-analysis” and “systematic review” up to December 2013.
2. Systematic reviews of studies of patient populations requiring prolonged (>48 hours) ICU admission were then streamed into one of three categories; systematic reviews containing studies in which there was no intervention, a non-antibiotic based intervention, or SDD as an antibiotic based intervention for the prevention of VAP. For the purpose of this study, SDD was factorized into protocolized topical and protocolized parenteral antibiotic prophylaxis (PPAP) components. An SDD study is defined here as the use of protocolized topical antibiotic prophylaxis applied by the gastric or oro-pharyngeal route in the intervention group with or without the additional use of PPAP.
3. The studies were screened against the following eligibility criteria. Inclusion criteria; Bacteremia incidence data for all bacteremias totalled and extractable as an incidence proportion per patient. Exclusion criteria; studies limited to patients with the acute respiratory distress syndrome. Studies in a language other than English were included when the required data had been abstracted in an English language systematic review.
4. A hand search was undertaken for additional studies meeting the eligibility criteria.
5. All eligible studies were then collated and any duplicate studies were removed. Ineligible studies that were not evaluable for the bacteremia end point but evaluable within a sensitivity analysis or for bacteremia isolate data were identified.
6. Groups of patients receiving mechanical ventilation from studies without a VAP prevention method under study were labelled as observational groups (Figure 2). The studies of intervention studies were classified as follows. Among the non-antibiotic based methods of VAP prevention are studies with interventions delivered at either the gastric site or the airway or oral sites. The SDD studies were further sub-classified (Figure 3); firstly as to whether the control group was concurrent and co-located within the same ICU as the intervention group (RCCT; Figure 3b) or not (non-concurrent; Figure 3a); and secondly on the basis of the additional use or not of PPAP in either the intervention group or the control group. Studies that used PPAP in the control group are referred to as duplex studies (Figure 3c).

7. The component groups were decanted from each study as follows;

The control and intervention groups from non-antibiotic based methods were classified as indicated in the original study

All groups that received topical antibiotic prophylaxis with or without PPAP were designated as an SDD intervention group and all other groups from SDD studies are classified as a control group regardless of whether or not they may have received PPAP (duplex studies).

### Data extraction

The primary outcome here is the bacteremia incidence proportion per 100 patients (B-IP) for each identified component group. Any studies with bacteremia incidences expressed only as a number of episodes or on a per-day basis were non evaluable for the primary outcome. Studies that reported bacteremia incidence proportion using a composite total of specific bacteremia sub-types were analysed here within a sensitivity analysis only. Coagulase negative Staphylococci (CNS) are common among bacteremia isolates of patients receiving SDD. Hence the defining criteria for bacteremia for each study was determined in relation to whether or not the CDC criteria for a Coagulase negative Staphylococcal isolate being detected in two or more separate blood cultures was specified [125].

For many SDD studies, but not all, the primary end point was VAP occurrence whereas bacteremia was a secondary end point. Hence for this analysis, the VAP incidence proportion per 100 patients (VAP-IP) was also extracted and analysed in parallel to enable an assessment of comparability between the end points of SDD studies selected here which had B-IP data available versus the broader evidence base for which B-IP data was not available.

The following bacteremia isolate data was extracted; numbers of coagulase negative Staphylococci (CNS); numbers of *Pseudomonas aeruginosa* isolates and total numbers of bacteraemia isolates.

### Statistical analysis

The bacteremia data were logit transformed for analysis as previously [122]; with the total number of patients as the denominator (D), the number of patients with bacteremia as the numerator (N), and R being the B-IP proportion (N/D), the logit(bacteremia-IP) is log(N/(D-N)) and its variance is 1/(D*R*(1-R)) [126]. Using these pre-calculated logits and logit variances, group specific 95% confidence intervals, summary logits and the associated summary 95% CIs were generated using the ‘metan’ command in STATA (release 12.0, STATA Corp., College Station, TX, USA) [127],[128].

For each category of component group the summary mean logit B–IP and associated 95% confidence interval were calculated using random effects methods. These were then back-transformed to the percentage scale. On the logit scale the 95% confidence intervals for a proportion are symmetrical and remain within the interval of 0 to 100%. The summary mean B–IP derived from the observational studies is the benchmark. The caterpillar plots have the studies ranked in order of increasing B-IP in relation to the benchmark.

VAP-IP data per total number of patients and the bacteremia isolate data per total number of isolates were likewise logit transformed to enable the analysis of these data.

A conventional meta-analysis of effect of study intervention was undertaken to derive study specific and summary measures using random effects meta-analysis methods and expressed as an odds ratio and displayed in forest plots. These summary measures derived from the category of non-antibiotic methods include a broad category of heterogenous interventions and the summary measures derived here are merely indicative. Caterpillar plots are forest plots which have been transformed by the ranking of studies in order of increasing study or group specific effect size.

Contextual effects of control and intervention group membership were each estimated in separate models versus the benchmark groups as the reference category using random effects meta-regression of group level logit transformed data. The use of CDC defining criteria for bacteremia and use of SDD factorized as topical and or parenteral antibiotic use and were entered as group level variables.

### Sensitivity analyses

Three sensitivity tests were undertaken to test the robustness of the findings here as follows; to the inclusion of additional data from studies that might be unpublished or missing; to the exclusion of groups from studies not cited in systematic reviews; and also to the inclusion of study results from two recent large cluster randomized studies of SDD and SOD [60],[63]. In sensitivity test one, the control groups from studies of non-antibiotic methods were used as a source of simulated ‘missing’ data in the recalculation of mean B-IP. In sensitivity test two, the meta-regression model for observational and control groups was repeated with restriction to only those component group obtained from studies sourced exclusively from systematic reviews. In sensitivity test three, the meta-regression model for observational and intervention groups was repeated including the bacteremia incidence data for intervention groups obtained from the two recent large cluster randomized studies of SDD and SOD. For the purpose of sensitivity test three the non-standard bacteremia definition as reported in these two studies was included in the meta-regression model after transformation to the logit scale but otherwise without adjustment.

## Results

### Description of studies

Of the 112 studies (Figure 1; Additional file 1: Tables S1-S5), 71 were sourced from at least one of 20 systematic reviews and 41 were sourced from elsewhere. Six were not evaluable for bacteremia incidence; two reported incidence for only specific bacteremia sub-types [64],[74], two reported an incidence of several specific bacteremia sub-types as a composite [60],[63] and two reported numbers of bacteremia episodes [69],[86]. The data from these six studies was analysed either only for bacteremia isolate data or only within sensitivity test three.

Of the 106 remaining studies, the CDC defining criteria for bacteremia were used in 49 studies. Following the decant of groups from the studies, there were 39 observational groups, 69 control and 77 intervention groups (Table 1). 13 studies either had a second control group or a second intervention group. Most studies were published in the 1990s. The SDD studies tended to be smaller in size and all but three were of European origin. There were 25 different antibiotic regimens under study in the 55 SDD intervention groups. The study groups were classified according to the type of study design in which they were located (Figures 2 & 3). All of the studies of non-antibiotic methods were concurrent in design. There were 13 control groups (duplex studies) and 40 intervention groups in which all patients received protocolized parenteral antibiotic prophylaxis (PPAP).

**Table 1:** **Characteristics of studies** ^a^

	Observational studies	Groups of interventional studies of VAP prevention				
		Non-antibiotic methods		Antibiotic (SDD)		
		Gastric	Airway or oral	Non-concurrent	RCCT	RCCT -Duplex
Study characteristics						
Sources [see Additional file 1]	Table S1	Table S2	Table S2	Table S3	Table S4	Table S5
Number of studies	36	13	9	16	28	12
Non-SR	29	5	3	7	2	0
EU origin^b^	18	6	8	8	20	8
MV for >48 hours for <90%^c^	12	2	1	5	5	0
Trauma ICUs^d^	2	2	1	1	5	3
CDC^e^	23	4	4	6	14	5
Study publication year (range)	1987-2013	1989-2014	1993-2013	1987-2011	1988-2007	1990-2002
Group characteristics						
Numbers of patients per study group; median (IQR)^f^	302; 153-846	74; 39-156	132; 76-190	104; 48-161	54; 39-110	43; 38-58
VAP incidence per 100 patients;						
mean; 95% CI (number of groups)						
Observational	22.2%; 17.8–27.3% (31)^g^	NA		NA	NA	
Control	NA	21.9%; 14.4-31.7% (12) ^h^	17.1%; 8.9-30.4% (8)^h^	36.8%; 16.7-62.8% (10)^i^	31.2%; 21.7-42.5% (24)^i^	22.7%; 14.4-34.2% (11)^i^
Intervention	NA	14.0%; 9.0-21.2% (13) ^h^	11.7%; 6.0-21.3% (9)^h^	13.1%; 6.2 – 25.5 (13)^j^	12.4%; 8.9-17.0% (27)^j^	7.1%; 3.5-13.8% (10)^j^
Bacteremia incidence per 100 patients;						
mean; 95% CI (number of groups)						
Observational	8.3%; 6.8–10.2% (39)^k^	NA	NA	NA	NA	NA
Control	NA	7.1%; 4.1-12.2% (12)^l,m,n^	7.1%; 3.5-14.1% (8)^l,m,n^	12.0%; 6.9-20.2% (10)^o^	17.1%; 13.1-22.1% (27)^o,n^	NA
Control - duplex						5.7%; 3.1-10.8% (13)^o^
Intervention – non-antibiotic	NA	5.8%; 3.3-9.9% (13)^l^	4.9%; 2.2-10.4% (9)^l^			
Intervention – topical antibiotic alone				12.8%; 7.8-20.5% (3)^p^	16.2%; 9.1-27.3% (12)^p^	NA
Intervention – topical and parenteral antibiotic				6.3%; 3.9-10.1% (10)^p^	8.9%; 5.8-13.3% (18)^p^	7.7%; 4.8-12.2% (13)^p^
Bacteremia microbiology per 100 isolates^q^						
mean; 95% CI (number of groups)						
• Coagulase negative Staphylococci						
Observational	16.2%; 12.1-21.2% (16)^q,r^	NA	NA	NA	NA	NA
Control	NA	NA	NA	3.7%; 0.2-39.3% (1)^q,s^	32.6%; 23.0-44.0% (5)^q,s^	27.5%; 12.1-50.9% (5)^q,s^
• *Pseudomonas aeruginosa*						
Observational	7.7%; 6.2–9.4% (16)^q,t^	NA	NA	NA	NA	NA
Control	NA	NA	NA	7.7%; 1.1-39.1% (1)^q,u^	5.6%; 2.7-11.3% (7)^q,u^	10.6%; 2.7-34.2% (3)^q,u^

^a^Abbreviations; ICU, Intensive care unit; MV; EU, European Union; Mechanical ventilation; NA not applicable; RCCT randomized concurrent controlled trials.

^b^Originating from a member state of the EU as at 2010 or Switzerland or Norway.

^c^Studies for which less than 90% of patients were reported to receive more than 48 hours of mechanical ventilation.

^d^Trauma ICU defined as an ICU with >50% of patient admissions for trauma.

^e^Studies that indicated that CDC criteria were used to define bacteremia.

^f^Data is median and inter-quartile range (IQR).

^g^As derived in Figure S1 (see Additional file 2).

^h^As derived in Figure S2 (see Additional file 2).

^i^As derived in Figure S3 (see Additional file 2).

^j^As derived in Figure S4 (see Additional file 2).

^k^As derived in Figure 4.

^l^As derived in Figure 5.

^m^The bacteremia incidence on inclusion of all concurrent control groups from studies of non-antibiotic methods is 7.1; 4.8-10.5 (n = 20).

^n^Sensitivity test one. The bacteremia incidence on inclusion of all concurrent control groups not receiving PPAP from studies of non-antibiotic and antibiotic methods is 11.9; 9.3-15.3 (n = 47).

^o^As derived in Figure 6.

^p^As derived in Figure 7.

^q^See Table S6 (Additional file 1).

^r^As derived in Figure S5 (see Additional file 2).

^s^As derived in Figure S6 (see Additional file 2).

^t^As derived in Figure S7 (see Additional file 2).

^u^As derived in Figure S8 (see Additional file 2).

VAP-IP data was available for 158 groups (Additional files 1 and 2: Figures S1-4) and bacteremia-IP data was available for 186 groups (Additional file 1; Figures 4, 5, 6 and 7).

Among all SDD studies with a concurrent design, the effect size expressed as an odds ratio for the difference for VAP incidences between control and intervention groups was 0.36 (0.31 – 0.42; n = 35) and for bacteremia incidences was 0.69 (0.59 – 0.81; n = 41). Among all ten SDD studies with a non-concurrent design, the effect size expressed as an odds ratio for the difference for VAP incidences between control and intervention groups was 0.50 (0.41 – 0.61) for bacteremia incidences was 0.75 (0.51– 0.99), respectively (Forest plots showing summary and study specific effect sizes are displayed in Additional file 3).

**Figure 4:**
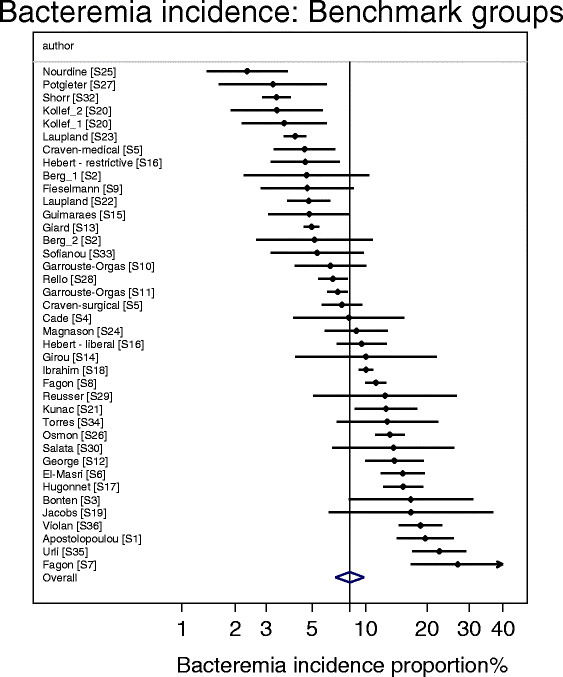
**Caterpillar plots of the group specific (small diamonds) and summary (large open diamond, vertical line) bacteremia incidence proportion (B-IP) and 95% CI of observational groups of observational studies (Benchmark groups).** Studies are listed in Additional file 1: Table S1. Note that the x axis is a logit scale.

**Figure 5:**
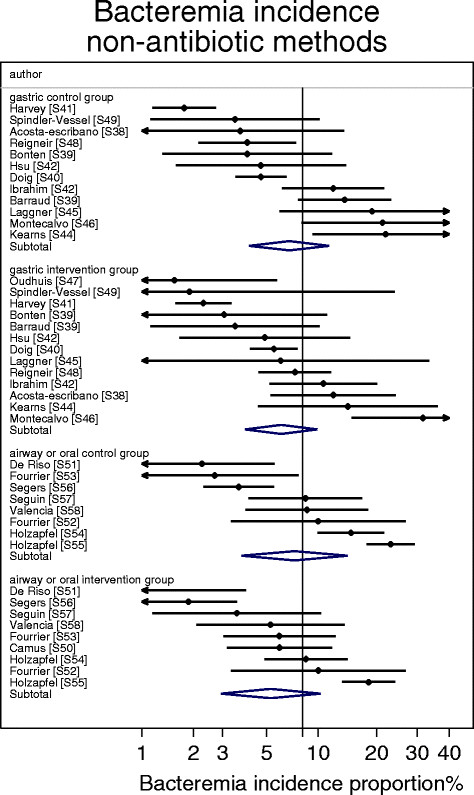
**Caterpillar plots of the group specific (small diamonds) and summary (large open diamond) B-IP and 95% CI of control and intervention groups from studies of VAP prevention using non-antibiotic methods.** For comparison, the summary B-IP (vertical line) derived from the benchmark groups from Figure 4 is shown. Studies are listed in Additional file 1: Table S2. Note that the x axis is a logit scale.

**Figure 6:**
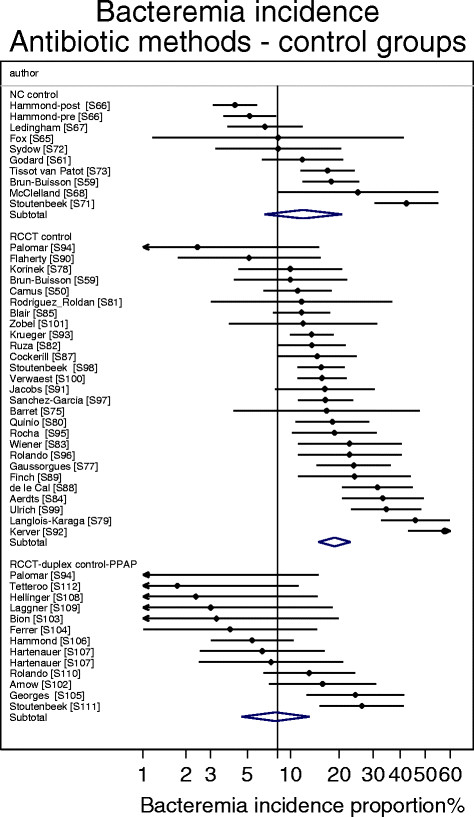
**Caterpillar plots of the group specific (small diamonds) and summary (large open diamond) B-IP and 95% CI of control groups of studies of VAP prevention using SDD.** Duplex study control groups received protocolized parenteral antibiotic prophylaxis. For comparison, the summary B-IP (vertical line) derived from the benchmark groups from Figure 4 are shown. Studies are listed in Additional file 1: Tables S3, S4 and S5. Note that the x axis is a logit scale.

**Figure 7:**
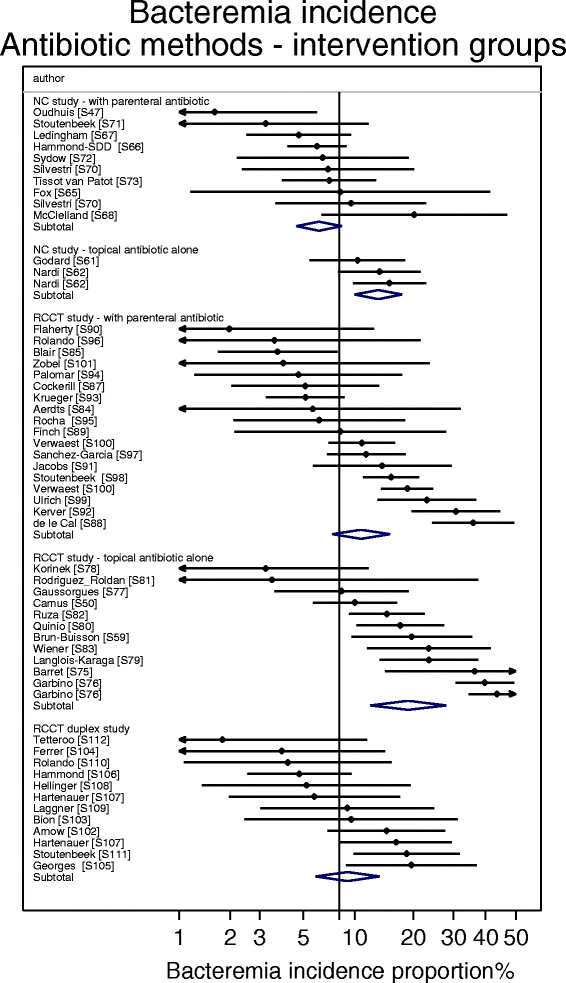
**Caterpillar plots of the group specific (small diamonds) and summary (large open diamond) B-IP and 95% CI of intervention groups of studies of VAP prevention using SDD.** For comparison, the summary B-IP (vertical line) derived from the benchmark groups from Figure 4 are shown. Studies are listed in Additional file 1: Tables S3, S4 and S5. Note that the x axis is a logit scale.

The bacteremia incidence benchmark was 8.3 (6.8-10.2) (Table 1; Figure 4). The mean bacteremia-IP for each of the four categories of component group from studies of non-antibiotic methods were within 4 percentage points of the bacteremia incidence benchmark (Table 1; Figure 5). Among the 8 categories of component group from the SDD studies, the mean bacteremia incidence among the four that received PPAP (three intervention and one control) were all also within 4 percentage points of the bacteremia incidence benchmark whereas three categories that did not receive PPAP (two intervention and one control groups) were > 4 percentage points greater than the benchmark (Table 1; Figures 6 and 7).

As a sensitivity test to groups from potentially unpublished or missing studies, the mean bacteremia incidence was re-calculated including all 46 concurrent control groups not receiving PPAP (i.e. the 20 control groups from studies of non-antibiotic based methods together with the 27 control groups from SDD studies not using PPAP) and this remains >1.5 percentage points greater than the upper 95% confidence limit of the mean B-IP of the benchmark (sensitivity test 1; Table 1, footnote n).

### Meta-regression models of logit B-IP

The effect of membership of the various categories of component group together with the effect of exposure to PPAP were examined in meta-regression models of logit B-IP separately for control and for intervention groups (Table 2). The effects of membership of either a control or an intervention group of an SDD RCCT were each significant, positive and similar in magnitude to the negative effect of exposure to PPAP on bacteremia incidence. The influences of all other factors in each model were non-significant.

**Table 2:** **Logit bacteremia-IP meta-regression models** ^**a**^

Factor	Coefficient ^b^	95% CI	p
**Observation and control groups**			
Groups from observational studies (reference group)	−2 · 26	−2 · 56 - -1 · 96	<0 · 001
Non-antibiotic methods;			
• Gastric study control	−0 · 28	−0 · 82 - +0 · 26	0 · 31
• Airway study control	−0 · 11	−0 · 72 - +0 · 49	0 · 71
SDD control groups;			
• Non-concurrent control	+0 · 43	−0 · 12 - +0 · 98	0 · 12
• RCCT control^c^	+0 · 90	+0 · 52 - +1 · 28	<0 · 001
• Control group receiving PPAP^d^	−0 · 94	−1 · 54 - -0 · 34	0 · 002
CDC bacteremia criteria	−0 · 24	−0 · 54 - +0 · 07	0 · 13
**Observation and intervention groups**			
Groups from observational studies (reference group)	−2 · 35	−2 · 64 - -2 · 07	<0 · 001
Non-antibiotic methods;			
• Gastric study intervention	−0 · 32	−0 · 84 - +0 · 21	0 · 23
• Airway study intervention	−0 · 39	−0 · 97 - +0 · 18	0 · 18
SDD intervention groups;			
• Non-concurrent and topical intervention alone^e^	+0 · 48	−0 · 12 - +1 · 08	0 · 12
• RCCT and topical intervention alone	+0 · 97	+0 · 50 - +1 · 44	<0 · 001
• Intervention group receiving PPAP^d^	−0 · 77	−1 · 24 - -0 · 29	0 · 002
CDC bacteremia criteria	−0 · 09	−0 · 38 - +0 · 21	0 · 56

^a^Abbreviations; ICU, Intensive care unit; RCCT, randomized concurrent control trial; TAP, topical antibiotic prophylaxis; PPAP, protocolized parenteral antibiotic prophylaxis.

^b^Interpretation. For each model the reference group is the observational study (benchmark) groups and this coefficient equals the difference in logits from 0 (a logit equal to 0 equates to a proportion of 50%; a logit equal to −2.40 equates to a proportion of 8.3%) and the other coefficients represent the difference in logits for groups positive for that factor versus the reference group.

^c^Sensitivity test two. Restriction of this meta-regression to component groups of studies sourced exclusively from systematic reviews yields a coefficient for control groups from SDD-RCCT’s remains positive and significantly different from zero (+0.89; +0.28 − +1.5; p = 0.005).

^d^PPAP is Protocolized parenteral antibiotic prophylaxis. As indicated in Figure 1, control groups in duplex studies and three categories of SDD intervention group received PPAP.

^e^Sensitivity test three. Inclusion of four intervention groups from two large cluster randomized studies that used non-standard composite definitions of bacteremia in this meta-regression yields a coefficient for intervention groups from non-concurrent studies of SDD or SOD which remains positive and non-significantly different from zero (+0.40; +0.17 - +0.96; p = 0.17).

Repeating the meta-regression model for observational and control groups with restriction to only those component group obtained from studies sourced exclusively from systematic reviews gave a coefficient that remained significant and positive (sensitivity test two; Table 2, footnote c).

The two large cluster randomized studies of SDD and SOD that had used a non-standard bacteremia end point, being the composite total of specific bacteremia sub-types, contained 1990 control group and 14940 intervention group patients [60],[63] versus the 4575 control group and 5238 intervention group patients from the 54 studies of SDD that had used a standard bacteremia end point, being incidence proportion totalled for all bacteremias. The meta-regression model for observational and intervention groups including unadjusted non-standard bacteremia end point data from the intervention groups from these two recent large cluster randomized studies of SDD and SOD was repeated. With the bacteremia data for these groups included in the model, the coefficient remained non-significant and positive (sensitivity test three; Table 2, footnote e)

### Bacteremia isolates

**Figure 8:**
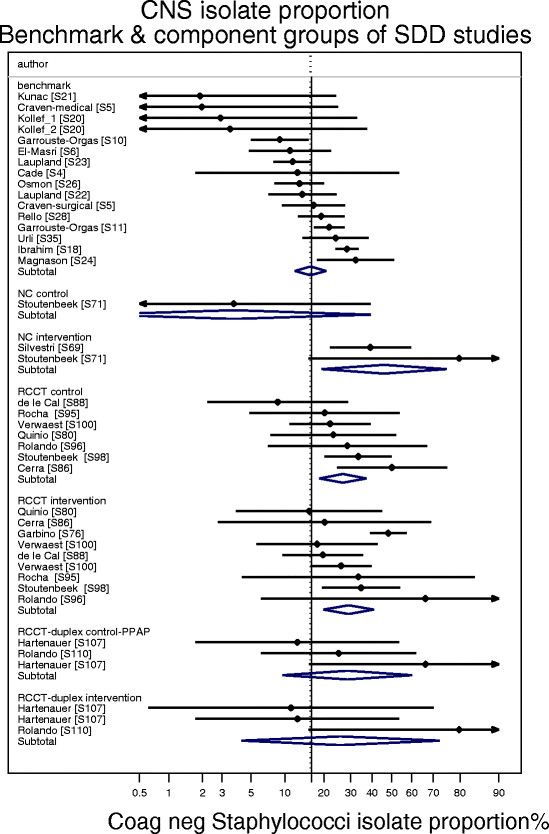
**Caterpillar plots of the group specific (small diamonds) and summary (large open diamond) coagulase negative Staphylococcus (CNS) as an isolate proportion (CNS-IP) and 95% CI of component groups of studies of VAP prevention using SDD and non-antibiotic methods.** The summary CNS-IP derived from the benchmark groups at the top of the figure is shown (vertical line). Note that the x axis is a logit scale. Studies are listed in Additional file 1: Tables S6.

**Figure 9:**
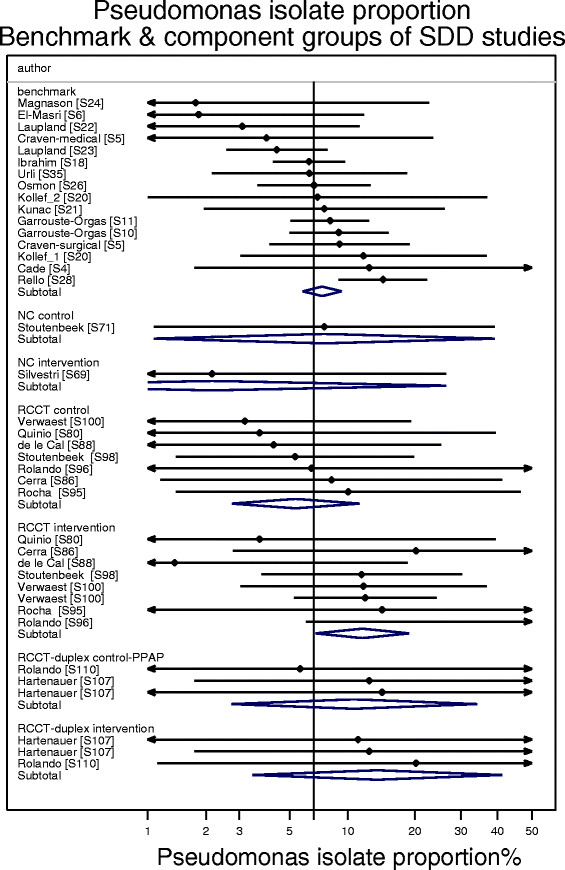
**Caterpillar plots of the group specific (small diamonds) and summary (large open diamond) Pseudomonas aeruginosa as an isolate proportion (Ps-IP) and 95% CI of component groups of studies of VAP prevention using SDD and non-antibiotic methods.** The summary Ps-IP derived from the benchmark groups at the top of the figure is shown (vertical line). Note that the x axis is a logit scale. Studies are listed in Additional file 1: Tables S6.

The proportion of CNS (Figure 8) and *Pseudomonas aeruginosa* (Figure 9) among bacteremia isolates was examined among groups from 15 benchmark and seven SDD-RCCT studies reporting bacteremia using the CDC criteria here (Table 1, Additional file 1: Tables S6). The proportion of CNS isolates among the control (p = 0.027) groups of the SDD-RCCT studies is double that versus the benchmark groups (Figure 8). By contrast, the proportion of *Pseudomonas aeruginosa* among the bacteremia isolates among the control groups of SDD-RCCT studies is similar to that of the benchmark groups (Figure 9; p = 0.64).

## Discussion

The effect of SDD on bacteremia is of great interest for five reasons. Bacteremia acquired by patients in the intensive care unit is associated with a high attributable mortality, especially so in patients either receiving mechanical ventilation [129] or who have pneumonia as the source of the bacteremia [129],[130].

Secondly, the defining criteria for bacteremia are less diverse than is the case for VAP. Hence bacteremia serves as a more stable study end point than is VAP toward estimating contextual effects [131]. Thirdly, SDD has complex ecological effects on colonization within the ICU [132] and clarifying the nature, direction and extent of these effects are crucial in defining the role of SDD going forward. Fourth, the numbers of patients assessed for a bacteremia end point in the SDD and SOD evidence has recently nearly doubled with the publication of two large cluster randomized studies [60],[63].

Finally, the effect of SDD on bacteremia within the ICU patient population is unclear and the evidence is conflicting. On the one hand, the evidence for protection against bacteremia [118],[120], as with protection against VAP, appears compelling [119],[120]. Among the 35 SDD RCCTs studies here, SDD appears to reduce bacteremia incidence by up to 31% and VAP by up to 64% [see Additional file 3: Figures S9 and S10]. Indeed the summary ORs derived elsewhere for SDD on both bacteremia among 31 studies [118] and pneumonia among 36 studies [119], are respectively similar to the counterfactual effect for each derived here among the SDD-RCCTs (see Additional file 3).

On the other hand, protection against bacteremia is unequal among the different types of SDD-RCCT’s. It is most apparent among the 18 SDD-RCCT’s for which the intervention groups received both topical and PPAP components of SDD. However, the protection appears marginal and non-significant among the remaining SDD-RCCT’s for which either the intervention groups received only the topical component of SDD and not the PPAP component or among the SDD-RCCT’s for which the control groups received the PPAP component (duplex studies) (Additional file 3: Figure S10).

Moreover, protection against bacteremia is not apparent in nationwide surveys. For example among 19 ICUs of Dutch hospitals the bacteremia rates are 5 versus 4 per 100 patient days for ICUs using versus not using SDD respectively [133]. Likewise, among >280,000 admission to 203 ICUs in the UK reporting data to the Intensive care National Audit and research center, unit acquired bacteremia occurred in 2.7 versus 2.8 percent of ICU admissions for nine ICUs that were using SDD versus 196 that were not [134]. Curiously, the nine ICUs using SDD includes three that were using SDD with a PPAP component for which the bacteremia rates was 0.1%. The bacteremia rate amongst the other six ICUs that were using SDD without a PPAP component is unknown but presumably higher than 2.7%.

The benchmark for bacteremia incidence derived here is 8.3%. The upward dispersion in bacteremia incidence among component groups from SDD RCCTs away from this benchmark is striking with all but 2 of the 27 control groups and all but 2 of 12 SDD intervention groups that did not receive PPAP being above this benchmark. This upward dispersion is apparent in the meta-regression models as positive coefficients in association with membership of either control or intervention groups of SDD studies versus the significant negative coefficient associated with exposure to the factorized PPAP component of SDD (Table 2).

The results here are in contrast with two cluster randomized trials of SDD and selective oropharyngeal decontamination (SOD) among up to 16 Dutch ICUs reported by de Smet [60] and Oostdijk [63]. However, there are two critical design aspects of these two studies which render comparisons with the findings of other studies difficult. Firstly, the VAP incidence was not reported. Secondly, a composite bacteremia end point including only five bacteremia sub-types not including Coagulase negative Staphylococci (CNS) was reported for these studies [60],[63]. As a consequence, neither the bacteremia incidence nor the counterfactual effect of SDD on bacteremia as conventionally defined is known for these studies. The inclusion of the bacteremia incidence data as reported from these two studies in a sensitivity analysis fails to change the findings here (sensitivity test three). Of note the incidence per 100 patients of bacteremia sub-types not including CNS was > 9 for the one control group and > 6 for four of eight SDD intervention groups for this [60],[63] and two other studies ([64],[74], Additional file 1: Table S3) that were otherwise not evaluable for the analysis of B-IP as a consequence of using non-standard bacteremia end-points.

Coagulase negative Staphylococci (CNS) typically account for 16%-25% of episodes of bacteremia using the CDC bacteremia definitions in series of ICU patients not receiving SDD [Additional file 3 Figure S5]. CNS bacteremia is not without risk for increased mortality and length of stay [114],[115],[135],[136]. CNS are common bacteremia isolates in this patient population but moreso among SDD recipients due to the selective effect of the SDD antibiotics. For example, among SDD recipients, 7 of 16 bacteremias in a Dutch series of 46 patients with sepsis syndrome [137], 52 of 108 bacteremia episodes in a Swiss ICU [76], 9 of 23 episodes in an Italian ICU [69], 54 of 115 episodes in a Viennese haematological ICU [138] and 9 of 26 episodes in another Dutch ICU [139] were CNS.

The finding of a higher proportion of CNS but not Pseudomonas among the bacteremia isolates of control groups is difficult to explain other than by a contextual effect associated with and arising out of the SDD intervention groups receiving the topical antibiotic component of SDD within studies of SDD which is inapparent at the level of each individual study [140].

There are several limitations of this analysis.

Only 64 of the 206 studies in the broader evidence base [122] from which these studies were derived had evaluable bacteremia incidence data available. However, the SDD summary counterfactual effects of the various interventions against both the VAP and bacteremia end points derived for the studies included here are similar to those derived elsewhere for a broader panel of studies [118]-[120]. Moreover, the summary VAP incidences here are also comparable to those in the larger panel. Also, the findings for bacteremia incidence here remain apparent in an analysis limited to studies extracted exclusively from systematic reviews (sensitivity test two).

The lack of observer blinding in some studies needs to be considered. Knowledge of treatment allocation may have influenced the taking of blood cultures to document bacteremia. Moreover, the empiric use of (non-protocolized) parenteral antibiotic therapy in each study is an important unknown as non-use may account for vulnerability at the individual level and contribute to the SDD contextual effect in the ICU at the group level in each study.

This contextual analysis is observational and is undertaken at the group level rather than the patient level. It was not possible to study the impact of unmeasured and unknown patient level risk factors for B-IP. However, the magnitude of such a putative patient level risk factor as a promoter of bacteremia incidence would need to be stronger than is magnitude of the group wide use of PPAP as a protector toward reducing bacteremia incidence (Table 2) and consistently so across all the studies and yet also be profoundly unevenly distributed, predominating in the groups of SDD RCCTs versus other groups within the broader evidence base to be able to account for the discrepancies noted here. Alternatively, there could be unpublished or missing SDD studies with control groups having a B-IP in the range of the studies of non-antibiotic methods to account for the discrepancies noted here (Table 1). As a sensitivity analysis there would need to be >20 of such studies with component groups with B-IP in the range of those seen among control groups of studies of non-antibiotic methods to rectify this discrepancy (sensitivity test one).

## Conclusions

Within SDD RCCTs, the mean bacteremia incidence among concurrent component groups not exposed to PPAP is double that of the benchmark bacteremia incidence. These observations are paradoxical, as with similar observations for VAP incidences among these studies [141]. Apart from major publication bias, or the effect of major and as yet unidentified and mal-distributed patient level risk factors for both VAP and bacteremia, these profound discrepancies indicate a major contextual hazard associated with the topical component of SDD on bacteremia within RCCT’s against which protocolized parenteral antibiotic partially mitigates. The safety of SDD within the ICU environment remains a concern and inapparent outbreaks remain a possible explanation for these observations within the SDD studies [140].

### Key messages

While SDD appears highly effective for infection prevention within the mechanically ventilated patient group, several paradoxical findings for the incidence and microbiology of the pneumonia end point among the SDD-RCCT studies imply a contextual hazard.

A bacteremia-IP benchmark derived from 39 non-intervention groups of mechanically ventilated patients is 8.3%.

Among SDD-RCCT studies, the mean bacteremia-IP for 27 control and 12 intervention groups that did not received protocolized parenteral antibiotic prophylaxis are each double the bacteremia-IP benchmark, respectively.

In meta-regression models, the magnitude and statistical significance of the positive effect associated with membership of either a control or an intervention group of an SDD-RCCT study on bacteremia-IP is similar to the magnitude of the negative effect associated with protocolized parenteral antibiotic prophylaxis.

These and other paradoxical discrepancies indicate a major contextual hazard associated with the topical component of SDD against which protocolized parenteral antibiotic partially mitigates.

## Author’s contributions

As sole author, JH produced the design of the study, performed the statistical analysis and wrote the manuscript. JH read and approved the final manuscript.

## Additional files

## Electronic supplementary material

Additional file 1: **VAP-IP and bacteremia-IP data for observational studies (Table S1), studies of non-antibiotic-based methods of VAP prevention (Table S2), studies of SDD– non-concurrent groups (Table S3), studies of SDD– RCCT’s (Table S4), studies of SDD-RCCT’s with Duplex design (Table S5), and Numbers of Coagulase negative Staphylococcus and** ***Pseudomonas*** **bacteremia isolates (Table S6).** (PDF 517 KB)

Additional file 2: Caterpillar plots for VAP-IP data (Figures S1-4). (PDF 26 KB)

Additional file 3: Forrest plots showing counterfactual effects as odds ratios between control versus intervention group VAP incidence (Figures S5, S7 & S9) and bacteremia incidence (Figures S6, S8 & S10) as study specific and summary effect sizes. (PDF 82 KB)

Below are the links to the authors’ original submitted files for images.

Authors’ original file for figure 1

Authors’ original file for figure 2

Authors’ original file for figure 3

Authors’ original file for figure 4

Authors’ original file for figure 5

Authors’ original file for figure 6

Authors’ original file for figure 7

Authors’ original file for figure 8

Authors’ original file for figure 9

## Abbreviations

ICU: Intensive care unit
MV: Mechanical ventilation
SDD: Selective digestive decontamination
SOD: Selective oro-pharyngeal decontamination
VAP: Ventilator associated pneumonia
VAP-IP: Ventilator associated pneumonia incidence proportion
B-IP: Bacteremia incidence proportion
PPAP: Protocolized parenteral antibiotic prophylaxis
