# Supplementary material for: Topical antibiotics as a major contextual hazard toward bacteremia within selective digestive decontamination studies: a meta-analysis
Source: BMC Infect Dis. 2014 Dec 31;14:714. doi: 10.1186/s12879-014-0714-x (PMC4300056; doi:10.1186/s12879-014-0714-x)
Supplement: Supplementary file 1 — Additional file 1: VAP-IP and bacteremia-IP data for observational studies (Table S1), studies of non-antibiotic-based methods of VAP prevention (Table S2), studies of SDD– non-concurrent groups (Table S3), studies of SDD– RCCT’s (Table S4), studies of SDD-RCCT’s with Duplex design (Table S5), and Numbers of Coagulase negative Staphylococcus and Pseudomonas bacteremia isolates (Table S6). (PDF 517 KB) [file 12879_2014_714_MOESM1_ESM.pdf]

Additional file 1: Table of contents

Table S1: Listing of observational studies (Benchmark groups) 2

Table S2: Listing of studies of non-antibiotic-based methods of VAP prevention 4

Table S3: Listing of studies of SDD– non-concurrent groups 6

Table S4: Listing of studies of SDD– RCCT’s 8

Table S5: Listing of studies of SDD-RCCT’s with Duplex design 11

Table S6: Numbers of Coagulase negative Staphylococcus and *Pseudomonas* bacteremia isolates 13

References S1-S113 14

Table S1: Observational studies (Benchmark groups) [a]

Study	Bacteremia definition [b]	Group size	VAP-IP (%)	B-IP (%)
Apostolopoulou et al 2003 [1 a]	NS	175	32	19
Berg et al 1995 [2]	CDC	123	33	4.9
Berg et al 1995 [2]	CDC	130	16.2	5.4
Bonten et al 1997 [3]	NS	42	25	17
Cade et al 1993 [4]	CDC	98	36	8.2
Craven et al 1988 [5]	CDC	526	17	4.8
Craven et al 1988 [5]	CDC	799	9	7.5
El-Masri et al 2004 [6]	CDC	361	NA	15.2
Fagon et al 1989 [7]	NS	52	9	27
Fagon et al 1996 [8 a]	CDC	1978	17	11
Fieselmann et al 1999 [9]	CDC	224	21	4.9
Garrouste-Orgeas et al 2000 [10]	CDC	291	NA	6.5
Garrouste-Orgeas et al 2006 [11]	CDC	3247	NA	7.1
George et al 1998 [12]	CDC	223	13	14
Giard et al 2008 [13]	NS	7236	13	5.2
Girou et al 2000 [14]	CDC	50	22	10
Guimaraes et al 2006 [15 a]	NS	278	38	5
Hébert et al 1999 [16]	NS	420	21	9.5
Hébert et al 1999 [16]	NS	418	21	7.2
Hugonnet et al 2004 [17]	CDC	432	24	15
Ibrahim et al 2000 [18]	CDC	4913	NA	10
Jacobs et al 1990 [19]	NS	24	54	17
Kollef et al 1997 [20 a]	CDC	353	12	3.7
Kollef et al 1997 [20 a]	CDC	327	7	3.4
Kunac et al 2013 [21]	CDC	206	29	13
Laupland et al 2002 [22]	CDC	1017	NA	4.4
Laupland et al 2004 [23]	CDC	4473	NA	4.2
Magnason et al 2008 [24]	CDC	280	7.5	8.9
Nourdine et al 1999 [25]	CDC	607	13	2.3

Study	Bacteremia definition	Group size	VAP-IP (%)	B-IP (%)
Osmon et al 2008 [26]	CDC	893	NA	13
Potgieter et al 1987 [27]	NS	250	20	3.2
Rello et al 1994 [28]	NS	1650	NA	6.7
Reusser et al 1989 [29]	NS	40	38	13
Salata et al 1987 [30]	NS	51	41	14
Shorr et al 2004 [31, 32]	NA	3502	20.5	3.3
Sofianou et al 2000 [33 a]	NS	198	34	5.6
Torres et al 1990 [34 a]	NS	78	24	13
Urli et al 2002 [35]	CDC	178	65	22
Violan et al 1998 [36 a]	NS	314	26	18

Table S1 footnotes

a. The indicated studies were derived from the following systematic reviews;

- Melsen WG, Rovers MM, Bonten MJM. Ventilator-associated pneumonia and mortality: A systematic review of observational studies. Crit Care Med 2009;37:2709–18.
- Safdar N, Dezfulian C, Collard HR, Saint S. Clinical and economic consequences of ventilator-associated pneumonia: a systematic review. Crit Care Med 2005;33:2184–93.
- Agrafiotis M, Siempos II, Ntaidou TK, Falagas ME. Attributable mortality of ventilator-associated pneumonia: a meta-analysis. Intern J Tub Lung Dis 2011;15:1154-1163.

b. Bacteremia defining criteria; NA, not applicable, NS, not specified; CDC, Center for disease control definitions.

Table S2: Studies of non-antibiotic-based methods of VAP prevention [a]

	Bacteremia definition [b]	Control groups				Intervention groups			
		Exposure [c]	Group size	VAP-IP (%)	B-IP (%)	Exposure [c]	Group size	VAP-IP (%)	B-IP (%)
**Gastric based interventions**									
Acosta-escribano et al 2010 [37 a]	CDC	G feed	54	57	3.7	SB feed	50	32	12
Barraud et al 2010 [38 a]	NS	SC	80	19	14	Probiotic	87	26	3.4
Bonten et al 1995 [39 a]	NS	SC	74	22	4.1	sucrulfate	67	22	3
Doig et al 2013 [40]	NS	EN	682	18	4.8	TPN	681	15	5.7
Harvey et al 2014 [41]	NS	EN	1197	12	2	TPN	1191	11	2
Hsu et al 2009 [42 a]	CDC	G feed	62	24	4.8	SB feed	59	9	5.1
Ibrahim et al 2002 [43]	NS	Early feed	75	49	12	Late feed	75	31	11
Kearns et al 2000 [44 a]	NS	G feed	23	13	22	SB feed	21	19	14
Laggner et al 1989 [45 a]	NS	ranitidine	16	13	19	sucrulfate	16	6	6.3
Montecalvo et al [46]	CDC	G feed	19	11	21	SB feed	19	0	32
Oudhuis et al [47 a ,d]	CDC					Probiotic	130	8	12
Reigneir et al [48]	NS	SC	222	16	4.1	RGV	227	17	7.5
Spindler-Vessel et al 2007 [49 a]	CDC	SC	87	38	3.4	Probiotic	26	15	0
**Airway and oral-care-based interventions**									
Camus et al 2005 [50 a,e]	CDC					Mup/Chlx	130	19	6.2
De Riso et al 1996[51 a]	CDC		180	5	2.2	Chlx	173	1.7	1
Fourrier et al 2000 [52 a]	CDC	SC	30	50	10	Chlx	30	17	10
Fourrier et al 2005 [53 a]	CDC	Placebo	114	11	2.6	Chlx	114	11	6.1
Holzapfel et al 1993 [54]	NS	NTI	149	11	15	OTI	151	6	8.6
Holzapfel et al 1999 [55 a]	NS	SC	200	26	23	sinus CT	199	19	18
Segers et al 2006 [56 a]	CDC		469	16	3.6	Chlx	485	9.3	1.9
Seguin et al 2013 [57 a]	NS	Placebo	72	28	8.5	PVI	78	31	3.5
Valencia et al [58 a]	NS	SC	69	15	8.7	CPC	73	15	5.5

Table S2 footnotes;

a. The indicated studies were derived from the following systematic reviews;

- Messori A, Trippoli S, Vaiani M, Gorini M, Corrado A. Bleeding and pneumonia in intensive care patients given ranitidine and sucralfate for prevention of stress ulcer: meta-analysis of randomised controlled trials. BMJ 2000;321:1103–1106.
- Huang J, Cao Y, Liao C, Wu L, Gao F. Effect of histamine-2-receptor antagonists versus sucralfate on stress ulcer prophylaxis in mechanically ventilated patients: a meta-analysis of 10 randomized controlled trials. Crit Care 2010;14:R194.
- Alhazzani W, Almasoud A, Jaeschke R, Lo BW, Sindi A, Altayyar S, Fox-Robichaud A. Small bowel feeding and risk of pneumonia in adult critically ill patients: a systematic review and meta-analysis of randomized trials. Crit Care 2013;17:R127;
- Chan EY, Ruest A, Meade MO, Cook DJ. Oral decontamination for prevention of pneumonia in mechanically ventilated adults: systematic review and meta-analysis. BMJ 2007;334:889–900;
- Labeau SO, Van de Vyver K, Brusselaers N, Vogelaers D, Blot SI. Prevention of ventilator-associated pneumonia with oral antiseptics: a systematic review and meta-analysis. Lancet Infect Dis 2011;11:845-854.
- Petrof EO, Dhaliwal R, Manzanares W, Johnstone J, Cook D, Heyland DK. Probiotics in the critically ill: a systematic review of the randomized trial evidence. Crit Care Med 2012;40:3290-3302.
- Liu KX, Zhu YG, Zhang J, Tao LL, Lee JW, Wang XD, Qu JM. Probiotics' effects on the incidence of nosocomial pneumonia in critically ill patients: a systematic review and meta-analysis. Crit Care 2012;16:R109.
- Melsen WG, Rovers MM, Bonten MJM. Ventilator-associated pneumonia and mortality: A systematic review of observational studies. Crit Care Med 2009;37:2709–18.
- Safdar N, Dezfulian C, Collard HR, Saint S. Clinical and economic consequences of ventilator-associated pneumonia: a systematic review. Crit Care Med 2005;33:2184–93.

b. Bacteremia defining criteria: NA, not applicable, NS, not specified; CDC, Center for disease control definitions.

c. Treatment abbreviations; G feed, feeding by the gastric route; SB feed, feeding by the small bowel route; EN, Enteral nutrition; TPN Total parenteral nutrition; SC, standard care; RGV, residual gastric volume monitoring; Mup, mupirocin nasal ointment; Chlx, chlorhexidene oral care; NTI, naso-tracheal intubation; OTI, oro-tracheal intubation; CPC, cuff pressure monitoring by automation.

d. Oudhuis et al [47]; Non-concurrent control group of this study received SDD and is classified as an intervention group in Table S3.

e. Camus et al [50]; Concurrent control group of this four arm study is classified in Table S4.

Table S3: Studies of antibiotic based methods of VAP prevention (= SDD) – non-concurrent groups [a]

	Bacteremia definition [b, c]	Control groups			Intervention groups			
		Group size	VAP-IP (%)	B-IP (%)	Antibiotic	Group size	VAP-IP (%)	B-IP (%)
**Non-concurrent groups; intervention groups received TAP alone [d]**								
Brun-Buisson et al 1989 [59 a]	CDC	124	15	18				
de Smet et al (SOD) 2009 [60 a,c]	Composite	1990	NA	9.3	PTA	1904	NA	6.5
Godard et al 1990 [62 a]	CDC	84	16	12	PT	97	2	10
Nardi et al 2001 [63]	NS				PTA	104	19	13
Nardi et al 2001 [63]	NS				PTAM	119	8	15
Oostdijk et al (SOD) 2014 [63 c]	Composite				PTA	5442	NA	5.9
**Non-concurrent groups; Intervention groups received both TAP and PPAP [d]**								
Cerda et al 2007 [64, c]	MRSA				PTA-Ctx	377		8.8
Cerda et al 2007 [64, c]	MRSA				PTAV-Ctx	165		1.1
de Smet et al (SDD) 2009 [60 a,c]	Composite				PTA-Ctx	2045	NA	4.3
Fox et al 1991 [65 a]	NS	12	66	8	PTA-Ctx	12	50	8
Hammond et al 1995 [66]	CDC	719	11	4.3	PTA-Ctx	403	16	6.2
Hammond et al 1995 [66]	CDC	406	14	5.4				
Ledingham et al 1988 [67 a]	NS	161	11	6.8	PTA-Ctx	163	2	4.9
McClelland et al 1990 [68 a]	NS	12	50	25	PTA-Ctx	15	7	20
Oostdijk et al (SDD) 2014 [63 c]	Composite				PTA-Ctx	5549	NA	4.6
Oudhuis et al 2011 [47 a,e]	CDC				PGA-Ctx	124	7	13
Silvestri et al 1999 [69, c]	CDC (PE)				PTA-Ctx	117	4.3	19.7
Silvestri et al 2004 [70]	CDC				PTA-Ctx	42	45	10
Silvestri et al 2004 [70]	CDC				PTAV-Ctx	42	52	7
Stoutenbeek et al 1984 [71 a]	CDC	59	59	42	PTA-Ctx	63	8	3.2
Sydow et al 1990 [72 a]	NS	48	75	8.3	PTA-Ctx	45	7	6.7
Tissot vanPatot et al 1996 [73]	CDC	142	63	14.8	PTA-Cef	142	20	6.5
Viviani et al 2005 [74, c]	MRSA				PTAV-Ctx	55	NA	7.3
Viviani et al 2005 [74, c]	MRSA				PTAV-Ctx	50	NA	8

Table S3 footnotes;

a. The indicated studies were derived from the following systematic reviews;

- Hurley JC. Prophylaxis with enteral antibiotics in ventilated patients: selective decontamination or selective cross-infection? Antimicrob Agents Chemother 1995;39:941-7.
- Pileggi C, Bianco A, Flotta D, Nobile CG, Pavia M. Prevention of ventilator-associated pneumonia, mortality and all intensive care unit acquired infections by topically applied antimicrobial or antiseptic agents: a meta-analysis of randomized controlled trials in intensive care units. Crit Care 2011;15:R155.
- Silvestri L, Van Saene HKF, Milanese M, Gregori D, Gullo A. Selective decontamination of the digestive tract reduces bacterial bloodstream infection and mortality in critically ill patients. Systematic review of randomized, controlled trials. J Hosp Infect 2007;65:187-203.
- Vandenbroucke-Grauls CM, Vandenbroucke JP. Effect of selective decontamination of the digestive tract on respiratory tract infections and mortality in the intensive care unit. Lancet 1991;338:859-862.

b. Bacteremia defining criteria: NA, not applicable; NS, not specified; CDC, Center for disease control definitions; MRSA, methicillin resistant Staphylococcus aureus.

c. The following studies were not evaluable for bacteremia incidence because of non-standard definitions of bacteremia;

- Cerda et al [64] and Viviani et al [74] reported only MRSA bacteremias
- de Smet et al [60] and Oostdijk et al [63] reported a composite of five bacteremia sub-types not including coagulase negative Staphylococcus.
- Silvestri et al [70] reported number of bacteremia episodes.

d. Abbreviations; TAP, topical antibiotic prophylaxis; PPAP, protocolized parenteral antibiotic prophylaxis

e. Oudhuis et al [47]; Non-concurrent intervention group of this study received probiotic and is classified as an intervention group in Table S2.

Table S4: Studies of antibiotic based methods of VAP prevention (= SDD) – RCCT's [a]

	Bacteremia definition [b]	Control groups			Intervention groups			
		Group size	VAP-IP (%)	B-IP (%)	Antibiotic [c]	Group size	VAP-IP (%)	B-IP (%)
**RCCT's: intervention groups received TAP alone [d]**								
Brun-Buisson et al 1989 [59 a]	CDC	50	12	10	PNeNa	36	8	19
Barret et al 2001 [75 a]	NS	12	NA	17	PTA	11	NA	36
Camus et al 2005 [50 a]	CDC	126	24	11	PT	130	12	10
Garbino et al 2002 [76]	CDC				PNVfluc	101	11	40
Garbino et al 2002 [76]	CDC				PNV	103	9	44
Gaussorgues et al 1991 [77 a]	NS	59	NA	24	PGVA	59	NA	8.5
Korinek et al 1993 [78 a]	CDC	60	42	10	PTAV	63	24	3.2
Langlois-Karaga et al 1995 [79]	NS	50	56	46	PGA	47	30	23
Quinio et al 1996 [80 a]	NS	72	51	18	PGA	76	25	17
Rodriguez_Roldan et al 1990 [81 a]	NS	17	65	12	PTNeA	14	29	0
Ruza et al 1998 [82 a]	CDC	110	NA	14	PTNy	116	NA	15
Wiener et al 1995 [83 a]	CDC	31	26	23	PGNy	30	27	23

Table S4: RCCT's (Continued)

	Bacteremia definition [b]	Control groups			Intervention groups			
		Group size	VAP-IP (%)	B-IP (%)	Antibiotic [c]	Group size	VAP-IP (%)	B-IP (%)
**RCCT's intervention groups received TAP and PPAP**								
Aerdts et al 1991 [84 a]	CDC	39	69	33	PNoA-Ctx	17	6	5.9
Blair et al 1991 [85 a]	NS	170	22	12	PTA-Ctx	161	7	3.7
Cerra et al 1995 [86 a, e]	CDC (pe)	21	71	57	Ny-No	25	48	20
Cockerill et al 1992 [87 a]	NS	75	5	15	PGNy-Ctx	75	4	5.3
de le Cal et al 2005 [88 a]	CDC	54	48	31	PTA-Ctx	53	34	36
Finch et al 1991 [89 a]	NS	25	29	24	PTA-Ctx	24	20	8.3
Flaherty et al 1990 [90 a]	NS	56	8.9	5.4	PGNy-Cef	51	2	2
Jacobs et al 1992 [91]	NS	46	9	16	PTA-Ctx	49	12	14
Kerver et al 1988 [92 a]	NS	47	85	57	PTA-Ctx	49	12	31
Krueger et al 2002 [93 a]	CDC	262	11	13	PG-Cip	265	3	5.1
Palomar et al 1997 [94 a]	CDC	42	50	2.4	PTA-Ctx	41	17	4.9
Rocha et al 1992 [95 a]	CDC	54	46	19	PTA-Ctx	47	15	6.4
Rolando et al 1993 [96 a]	CDC	31	36	23	PTA-Ctx	28	29	3.6
Sanchez-Garcia et al 1998 [97 a]	NS	140	41	16	PTA-Ctx	131	29	11.5
Stoutenbeek et al 2007 [98 a]	NS	200	23	16	PTA-Ctx	201	10	15.4
Ulrich et al 1989 [99 a]	NS	52	50	35	PNoA-Tr	48	15	23
Verwaest et al 1997 [100 a]	CDC	185	22	16	OA-O	193	11	11
Verwaest et al 1997 [100 a]	CDC				PTA-Ctx	200	16	19
Zobel et al 1991 [101]	NS	25	NA	12	PGA-Ctx	25	NA	4

Table S4 footnotes;

a. The indicated studies were derived from the following systematic reviews;

- Liberati A, D'Amico R, Pifferi S, Torri V, Brazzi L, Parmelli E. Antibiotic prophylaxis to reduce respiratory tract infections and mortality in adults receiving intensive care (Review). Cochrane Database Syst Rev 2009; CD000022.
- Pileggi C, Bianco A, Flotta D, Nobile CG, Pavia M. Prevention of ventilator-associated pneumonia, mortality and all intensive care unit acquired infections by topically applied

antimicrobial or antiseptic agents: a meta-analysis of randomized controlled trials in intensive care units. Crit Care 2011;15:R155.

- Silvestri L, Van Saene HKF, Milanese M, Gregori D, Gullo A. Selective decontamination of the digestive tract reduces bacterial bloodstream infection and mortality in critically ill patients. Systematic review of randomized, controlled trials. J Hosp Infect 2007; 65:187-203.

b. Bacteremia defining criteria: NA, not applicable, NS, not specified; CDC, Center for disease control definitions; pe per episode.

c. Treatment abbreviations

- letters before the hyphen referring to topical components; Pl, topical placebo; P, topical polymyxin; T, topical tobramycin; A, topical amphotericin; No, topical norlfoxacin; G, topical gentamicin; Cip, topical ciprofloxacin, V, topical vancomycin; Ny, topical nystatin; Ne, topical neomycin; Na, topical nalidixic acid; No, topical norlfoxacin; O, topical oflaxcin;
- letters after the hyphen refer to parenteral components; -Cef, Cefazolin; -Ctx, systemic cefotaxime; -Cip, systemic ciprofloxacin; -V, systemic vancomycin; -No, systemic norfloxacin; -Tr, systemic trimethoprim; -O, systemic oflaxcin; -Cz, systemic ceftazidime.

d. Abbreviations; TAP, topical antibiotic prophylaxis; PPAP, protocolized parenteral antibiotic prophylaxis

e. The following study was not evaluable because the bacteremia incidence was reported as number of bacteremia episodes; Cerra et al 1995 [86]

Table S5: Studies of antibiotic based methods of VAP prevention (= SDD) RCCT's - Duplex groups [a]

	Bacteremia definition [b]	Control groups				Intervention groups			
		Antibiotic [c]	Group size	VAP-IP (%)	B-IP (%)	Antibiotic [c]	Group size	VAP-IP (%)	B-IP (%)
**RCCT's: control groups received PPAP alone; intervention groups received TAP and PPAP [d]**									
Arnow et al [102 a]	CDC	Ctx	38	NA	12	PTNy-Ctx	48	NA	3
Bion et al 1991 [103 a]	NS	Ctx&Amp	31	26	3.2	PTA-Ctx	21	0	9.5
Ferrer et al 1994 [104 a]	NS	Ctx	50	24	4	PTA-Ctx	51	18	3.9
Georges et al 1994 [105 a]	NS	Amx-Clav	33	46	24	PNA-AmxClav	31	10	19
Hammond et al 1992 [106 a]	NS	Ctx	160	6	5.6	PTA-Ctx	162	7	4.9
Hartenaeur et al 1991 [107 a]	CDC	Ctx	61	46	6.6	PTA-Ctx	50	10	6
Hartenaeur et al 1991 [107 a]	CDC	Ctx	40	45	7.5	PTA-Ctx	49	10	16
Hellinger et al 2002 [108 a]	NS	Ctz	43	NA	2.3	PGNy-Ctx	37	NA	5.4
Laggner et al 1994 [109 a]	NS	Amx-Clav	34	12	2.9	GA-AmxClav	33	3	9.1
Palomar et al 1997 [94 a]	CDC	Ctx	46	30	0	PTA-Ctx	41	17	4.9
Rolando et al1996 [110 a]	CDC	CtzFlx	61	NA	13	PTA-CtzFlx	47	NA	4.3
Stoutenbeek et al 1996 [111 a]	NS	Ctx	42	19	26	PTA-Ctx	49	4	18
Tetteroo et al 1990 [112 a]	NS	Ctx	58	14	1.7	PTA-Ctx	56	2	1.8

Table S5 footnotes;

a. The indicated studies were derived from the following systematic reviews;

- Liberati A, D'Amico R, Pifferi S, Torri V, Brazzi L, Parmelli E. Antibiotic prophylaxis to reduce respiratory tract infections and mortality in adults receiving intensive care (Review). Cochrane Database Syst. Rev. 2009; CD000022;
- Chan EY, Ruest A, Meade MO, Cook DJ. Oral decontamination for prevention of pneumonia in mechanically ventilated adults: systematic review and meta-analysis. BMJ. 2007;334:889–900.
- Pileggi C, Bianco A, Flotta D, Nobile CG, Pavia M. Prevention of ventilator-associated pneumonia, mortality and all intensive care unit acquired infections by topically applied antimicrobial or antiseptic agents: a meta-analysis of randomized controlled trials in intensive care units. Crit Care 2011;15, R155.

- Melsen WG, Rovers MM, Bonten MJM. Ventilator-associated pneumonia and mortality: A systematic review of observational studies. Crit Care Med. 2009;37:2709–18.
- Safdar N, Dezfulian C, Collard HR, Saint S. Clinical and economic consequences of ventilator-associated pneumonia: a systematic review. Crit Care Med. 2005;33:2184–93
- Silvestri L, Van Saene HKF, Milanese M, Gregori D, Gullo A. Selective decontamination of the digestive tract reduces bacterial bloodstream infection and mortality in critically ill patients. Systematic review of randomized, controlled trials. J Hosp Infect 2007; 65:187-203.

b. Bacteremia defining criteria: NA, not applicable, NS, not specified; CDC, Center for disease control definitions.

c. Treatment abbreviations

   - letters before the hyphen referring to topical components; Pl, topical placebo; P, topical polymyxin; T, topical tobramycin; A, topical amphotericin; No, topical norlfoxacin; G, topical gentamicin; Cip, topical ciprofloxacin, V, topical vancomycin; Ny, topical nystatin; Ne, topical neomycin; Na, topical nalidixic acid; No, topical norlfoxacin; O, topical oflaxcin;
   - letters after the hyphen refer to parenteral components; - Amx-Clav, amoxicillin-clavulinate; -Ctx, systemic cefotaxime; -Cip, systemic ciprofloxacin; -V, systemic vancomycin; -Cip, systemic ciprofloxacin; -Tr, systemic trimethoprim; -O, systemic oflaxcin; -Cz, systemic ceftazidime.

d. Abbreviations; TAP, topical antibiotic prophylaxis; PPAP, protocolized parenteral antibiotic prophylaxis.

Table S6: Numbers of Coagulase negative Staphylococcus (CNS) bacteremia isolates [a]

Study	Group type[b]	Total number of bacteremia isolates	CNS isolates		Pseudomonas isolates	
			Number	Percent	Number	Percent
From Table S1						
Cade et al 1993 [4]	B	8	1	12.5	2	25
Craven et al 1988 [5]	B	25	0	0	1	4
Craven et al 1988 [5]	B	66	11	16.7	6	9
El-Masri et al 2004 [6]	B	40	6	15	1	3
Garrouste-Orgeas et al 2000 [10]	B	33	12	36.4	12	36
Garrouste-Orgeas et al 2006 [11]	B	232	50	21.6	19	8
Ibrahim et al 2000 [18]	B	339	96	28.3	22	6
Kollef et al 1997 [20]	B	14	0	0	2	14
Kollef et al 1997 [20]	B	9	0	0	1	11
Kunac et al 2013 [21]	B	26	0	0	2	8
Laupland et al 2002 [22]	B	66	9	14	2	3
Laupland et al 2004 [23]	B	244	28	11.4	11	5
Magnason et al 2008 [24]	B	28	9	32.1	0	0
Osmon et al 2008 [26]	B	131	17	13	9	7
Urli et al 2002 [35]	B	46	11	23.9	3	7
From Table S2						
Fourrier et al 2000 [52,]	C & I	6	2	33.3	0	0
Fourrier et al 2005 [53]	C & I	10	5	50	1	10
From Table S3						
Silvestri et al 1999 [69]	I	23	9	39.1	0	0
Stoutenbeek et al 1984 [71]	C	13	0	0	1	8
From Table S4						
Quinio et al 1996 [80]	C	13	3	23	0	0
Cerra et al 1995 [86]	C	12	6	50	1	8
de le Cal et al 2005 [88]	C	23	2	8.6	23	100
Rocha et al 1992 [95]	C	10	2	20	1	10
Rolando et al 1993 [96]	C	7	2	29	0	0
Stoutenbeek et al 2007 [98]	C	29	12	41	2	7
Verwaest et al 1997 [113]	C	32	7	21	1	3
Garbino et al 2003 [76]	I	108	52	48	NA	NA
Quinio et al 1996 [80]	I	13	2	15	0	0
Cerra et al 1995 [86]	I	6	1	17	1	17
de le Cal et al 2005 [88]	I	36	7	19.4	36	100
Rocha et al 1992 [95]	I	3	1	33	0	0
Rolando et al 1993 [96]	I	2	1	50	1	100
Stoutenbeek et al 2007 [98]	I	26	9	35	3	12
Verwaest et al 1997 [113]	I	17	3	18	2	12
Verwaest et al 1997 [113]	I	50	13	26	6	12

Table S6: Numbers of Coagulase negative Staphylococcus (CNS) bacteremia isolates (continued) [a]

Study	Group type[b]	Total number of bacteremia isolates	CNS isolates		Pseudomonas isolates	
			Number	Percent	Number	Percent
From Table S5						
Hartenaeur et al 1991 [107]	C	11	3	27	1	9
Hartenaeur et al 1991 [107]	I	7	1	14	1	14
Rolando et al1996 [110]	C	8	2	25	0	0
Rolando et al1996 [110]	I	2	2	100	0	0

Table S6 footnotes;

a. All studies used CDC defining criteria for bacteremia
b. Group type abbreviations; B benchmark; C control; I intervention; C & I control and intervention groups combined

References

1. Apostolopoulou E, Bakakos P, Katostaras T, Gregorakos L: **Incidence and risk factors for ventilator-associated pneumonia in 4 multidisciplinary intensive care units in Athens, Greece.** *Respir Care* 2003, **48**:681-688.
2. Berg DE, Hershow RC, Ramirez CA, Weinstein RA: **Control of nosocomial infections in an intensive care unit in Guatemala City.** *Clin Infect Dis* 1995, **21**:588-593.
3. Bonten MJ, Froon AH, Gaillard CA, Greve JWM, de Leeuw PW, Drent M, Buurman WA: **The systemic inflammatory response in the development of ventilator-associated pneumonia.** *Am J Respir Crit Care Med* 1997, **156**:1105-1113.
4. Cade JF, McOwat E, Siganporia R, Keighley C, Presneill J, Sinickas V: **Uncertain relevance of gastric colonization in the seriously ill.** *Intensive Care Med* 1992, **18**:210-7.
5. Craven DE, Kunches LM, Lichtenberg DA, Kollisch NR, Barry MA, Heeren TC, McCabe WR: **Nosocomial infection and fatality in medical and surgical intensive care unit patients.** *Arch Intern Med* 1988, **148**:1161-8.
6. El-Masri MM, Hammad TA, McLeskey SW, Joshi M, Korniewicz DM: **Predictors of nosocomial bloodstream infections among critically ill adult trauma patients.** *Infect Control Hosp Epidemiol* 2004, *25:*656-663.
7. Fagon JY, Chastre J, Domart Y, Trouillet JL, Pierre J, Darne C, Gibert C: **Nosocomial pneumonia in patients receiving continuous mechanical ventilation. Prospective analysis of 52 episodes**

with use of a protected specimen brush and quantitative culture techniques. *Am Rev Respir Dis* 1989, **139**:877-884.

8. Fagon JY, Chastre J, Vuagnat A, Trouillet JL, Novara A, Gibert C: **Nosocomial pneumonia and mortality among patients in intensive care units.** *JAMA* 1996, **275**:866-869.

9. Fieselmann JF, Bock MJ, Hendryx MS, Wakefield D, Helms CM, Bentler SE: **Mechanical ventilation in rural ICUs.** *Crit Care* 1999, **3**:23-31.

10. Garrouste-Orgeas M, Chevret S, Mainardi JL, Timsit JF, Misset B, Carlet J: **A one-year prospective study of nosocomial bacteraemia in ICU and non-ICU patients and its impact on patient outcome.** *J Hosp Infect* 2000, **44**:206-213.

11. Garrouste-Orgeas M, Timsit JF, Tafflet M, Misset B, Zahar JR, Soufir L, Carlet J: **Excess risk of death from intensive care unit—acquired nosocomial bloodstream infections: a reappraisal.** *Clin Infect Dis* 2006, **42**:1118-1126.

12. George DL, Falk PS, Wunderink RG, Leeper Jr KV, Meduri GU, Steere EL, Glen Mayhall C: **Epidemiology of ventilator-acquired pneumonia based on protected bronchoscopic sampling.** *Am J Respir Crit Care Med* 1998, **158**:1839-1847.

13. Giard M, Lepape A, Allaouchiche B, Guerin C, Lehot JJ, Robert MO, Vanhems P: **Early-and late-onset ventilator-associated pneumonia acquired in the intensive care unit: comparison of risk factors.** *J Crit Care* 2008, **23**:27-33.

14. Girou E, Schortgen F, Delclaux C, Brun-Buisson C, Blot F, Lefort Y, Brochard L: **Association of noninvasive ventilation with nosocomial infections and survival in critically ill patients.** *JAMA* 2000, **284**:2361-2367.

15. Guimaraes MM, Rocco JR: **Prevalence of ventilator-associated pneumonia in a university hospital and prognosis for the patients affected.** *J Bras Pneumol* 2006, **32**:339–346.

16. Hébert PC, Wells G, Blajchman MA, Marshall J, Martin C, Pagliarello G, Yetisir E: **A multicenter, randomized, controlled clinical trial of transfusion requirements in critical care.** *New Engl J Med* 1999, **340**:409-417.

17. Hugonnet S, Eggimann P, Borst F, Maricot P, Chevrolet JC, Pittet D: **Impact of ventilator-associated pneumonia on resource utilization and patient outcome.** *Infect Control Hosp Epidemiol* 2004, **25**:1090-6.

18. Ibrahim EH, Ward S, Sherman G, Kollef MH: **A comparative analysis of patients with early-onset vs late-onset nosocomial pneumonia in the ICU setting.** *Chest* 2000, **117**:1434-1442.

19. Jacobs S, Chang RW, Lee B, Bartlett FW: **Continuous enteral feeding: a major cause of pneumonia among ventilated intensive care unit patients.** *JPEN J Parenter Enteral Nutr* 1990, **14**:353-6.

20. Kollef MH, Vlasnik J, Sharpless L, Pasque C, Murphy D, Fraser V: **Scheduled change of antibiotic classes A strategy to decrease the incidence of ventilator-associated pneumonia.** *Am J Respir Crit Care Med* 1997, **156**:1040–1048.

21. Kunac A, Sifri ZC, Mohr AM, Horng H, Lavery RF, Livingston DH: **Bacteremia and Ventilator-Associated Pneumonia: A Marker for Contemporaneous Extra-Pulmonic Infection.** *Surgical Infections* 2014, **15**:77-83.

22. Laupland KB, Zygun DA, Davies HD, Church DL, Louie TJ, Doig CJ: **Population-based assessment of intensive care unit-acquired bloodstream infections in adults: incidence, risk factors, and associated mortality rate.** *Crit Care Med* 2002, **30**:2462-2467.

23. Laupland KB, Kirkpatrick AW, Church DL, Ross T, Gregson DB: **Intensive-care-unit-acquired bloodstream infections in a regional critically ill population.** *J Hosp Infect* 2004, *58*(2), 137-145.

24. Magnason S, Kristinsson KG, Stefansson T, Erlendsdottir H, Jonsdottir K, Kristjansson M, Gudmundsson S: **Risk factors and outcome in ICU‐acquired infections.** *Acta Anaesthesiologica Scandinavica* 2008, 52:1238-1245.

25. Nourdine K, Combes P, Carton MJ, Beuret P, Cannamela A, Ducreux JC : **Does noninvasive ventilation reduce the ICU nosocomial infection risk? A prospective clinical survey.** *Intensive Care Med* 1999, **25**:567-573.

26. Osmon S, Warren D, Seiler SM, Shannon W, Fraser VJ, Kollef MH: **The influence of infection on hospital mortality for patients requiring >48 h of intensive care.** *Chest* 2003, **124**:1021-1029.

27. Potgieter PD, Linton DM, Oliver S, Forder AA: **Nosocomial infections in a respiratory intensive care unit.** *Crit Care Med* 1987, **15**:495-498.

28. Rello J, Ricart M, Mirelis B, Quintana E, Gurgui M, Net A, Prats, G: **Nosocomial bacteremia in a medical-surgical intensive care unit: epidemiologic characteristics and factors influencing mortality in 111 episodes.** *Intensive Care Med* 1994, **20**:94-98.

29. Reusser P, Zimmerli W, Scheidegger D, Marbet GA, Buser M, Gyr K: **Role of gastric colonization in nosocomial infections and endotoxemia: a prospective study in neurosurgical patients on mechanical ventilation.** *J Infect Dis* 1989, **160**:414-421.

30. Salata RA, Lederman MM, Shlaes DM, Jacobs MR, Eckstein E, Tweardy D, Toossi Z, Chmielewski R, Marino J, King CH: **Diagnosis of nosocomial pneumonia in intubated, intensive care unit patients.** *Am Rev Respir Dis* 1987, **135**:426-432.

31. Shorr AF, Duh MS, Kelly KM, Kollef MH: **Red blood cell transfusion and ventilator-associated pneumonia: a potential link?** *Crit Care Med* 2004, **32**:666-674.

32. Shorr AF, Jackson WL, Kelly KM, Fu M, Kollef MH: **Transfusion Practice and Blood Stream Infections in Critically Ill Patients.** *Chest* 2005, **127**:1722-1728.

33. Sofianou DC, Constandinidis TC, Yannacou M, Anastasiou H, Sofianos E: **Analysis of risk factors for ventilator-associated pneumonia in a multidisciplinary intensive care unit.** *Eur J Clin Microbiol Infect Dis* 2000, **19**:460-463.

34. Torres A, Aznar R, Gatell JM, Jimenez P, Gonzalez J, Ferrer A, Celis R, Rodriguez-Roisin R: **Incidence, risk, and prognosis factors of nosocomial pneumonia in mechanically ventilated patients.** *Am Rev Respir Dis* 1990, **142**:523-528.

35. Urli T, Perone G, Acquarolo A, Zappa S, Antonini B, Ciani A: **Surveillance of infections acquired in intensive care: usefulness in clinical practice.** *J Hosp Infect* 2002, **52**:130-5.

36. Violan JS, Sanchez-Ramirez C, Mujica AP, Cendrero JC, Fernandez JA, de Castro FR; **Impact of nosocomial pneumonia on the outcome of mechanically-ventilated patients.** *Crit Care (Lond)* 1998, **2**:19-23.

37. Acosta-Escribano J, Fernández-Vivas M, Carmona TG, Caturla-Such J, Garcia-Martinez M, Menendez-Mainer A, Sanchez-Payá J: **Gastric versus transpyloric feeding in severe traumatic brain injury: a prospective, randomized trial.** *Intensive Care Med* 2010, **36**:1532-1539.

38. Barraud D, Blard C, Hein F, Marçon O, Cravoisy A, Nace L, Alla F, Bollaert PE, Gibot S; **Probiotics in the critically ill patient: a double blind, randomized, placebo-controlled trial.** *Intensive Care Med* 2010, **36**:1540-1547.

39. Bonten MJ, Gaillard CA, Van der Geest S, Van Tiel FH, Beysens AJ, Smeets HG, Stobberingh EE: **The role of intragastric acidity and stress ulcus prophylaxis on colonization and infection in mechanically ventilated ICU patients. A stratified, randomized, double-blind study of sucralfate versus antacids.** *Am J Respir Crit Care Med* 1995, **152**:1825-1834.

40. Doig GS, Simpson F, Sweetman EA, Finfer SR, Cooper DJ, Heighes PT, Peake S: **Early parenteral nutrition in critically ill patients with short-term relative contraindications to early enteral nutrition: a randomized controlled trial.** *JAMA* 2013, **309**:2130-2138.

41. Harvey SE, Parrott F, Harrison DA, Bear DE, Segaran E, Beale R, Bellingan G, Leonard R, Mythen MG, Rowan KM: **Trial of the Route of Early Nutritional Support in Critically Ill Adults**. *New England Journal of Medicine* 2014, 371:1673-1684.

42. Hsu CW, Sun SF, Lin SL, Kang SP, Chu KA, Lin CH, Huang HH: **Duodenal versus gastric feeding in medical intensive care unit patients: a prospective, randomized, clinical study.** *Crit Care Med* 2009, **37**:1866-1872.

43. Ibrahim EH, Mehringer L, Prentice D, Sherman G, Schaiff R, Fraser V, Kollef MH: **Early versus late enteral feeding of mechanically ventilated patients: Results of a clinical trial.** *JPEN J Parenter Enteral Nutr* 2002, **26**:174–181.

44. Kearns PJ, Chin D, Mueller L, Wallace K, Jensen WA, Kirsch CM: **The incidence of ventilator-associated pneumonia and success in nutrient delivery with gastric versus small intestinal feeding: a randomized clinical trial.** *Crit Care Med* 2000, **28**:1742-1746.

45. Laggner AN, Lenz K, Base W, Druml W, Schneeweiss B, Grimm G: **Prevention of upper gastrointestinal bleeding in long-term ventilated patients. Sucralfate versus ranitidine.** *Am J Med* 1989, **86**:81-4.

46. Montecalvo MA, Steger KA, Farber HW: **Nutritional outcome and pneumonia in critical care patients randomized to gastric versus jejunal tube feedings. The Critical Care Research Team.** *Crit Care Med* 1992, **20**:1377-1387.

47. Oudhuis GJ, Bergmans DC, Dormans T, Zwaveling JH, Kessels A, Prins MH, Verbon A: **Probiotics versus antibiotic decontamination of the digestive tract: infection and mortality.** *Intensive Care Med* 2011, **37**:110-117.

48. Reignier J, Mercier E, Le Gouge A, Boulain T, Desachy A, Bellec F, Lascarrou JB: **Effect of Not Monitoring Residual Gastric Volume on Risk of Ventilator-Associated Pneumonia in Adults Receiving Mechanical Ventilation and Early Enteral Feeding. A Randomized Controlled Trial.** *JAMA* 2013, **309**;249-256.

49. Spindler-Vesel A, Bengmark S, Vovk I, Cerovic O, Kompan L: **Synbiotics, prebiotics, glutamine, or peptide in early enteral nutrition: a randomized study in trauma patients.** *J Parenter Enteral Nutr* 2007, **31**:119-126.

50. Camus C, Bellissant E, Sebille V, Perrotin D, Garo B, Legras A, Renault A, Le Corre P, Donnio PY, Gacouin A: **Prevention of acquired infections in intubated patients with the combination of two decontamination regimens.** *Crit Care Med* 2005, **33**:307-314.

51. DeRiso AJ, Ladowski JS, Dillon TA, Justice JW, Peterson AC: **Chlorhexidine gluconate 0.12% oral rinse reduces the incidence of total nosocomial respiratory infection and nonprophylactic systemic antibiotic use in patients undergoing heart surgery.** *Chest* 1996, **109**:1556-1561.

52. Fourrier FE, Cau-Pottier H, Boutigny M, Roussel-Delvallez M, Jourdain, Chopin C: **Effects of dental plaque antiseptic decontamination on bacterial colonization and nosocomial infections in critically ill patients.** *Intensive Care Med* 2000, **26**:1239-1247.

53. Fourrier F, Dubois D, Pronnier P, Herbecq P, Leroy O, Desmettre T, Roussel-Delvallez M: **Effect of gingival and dental plaque antiseptic decontamination on nosocomial infections acquired in the intensive care unit a double-blind placebo-controlled multicenter study.** *Crit Care Med* 2005, **33**:1728-1735.

54. Holzapfel L, Chevret S, Madinier G, Ohen F, Demingeon G, Coupry A, Chaudet M: **Influence of long-term oro- or nasotracheal intubation on nosocomial maxillary sinusitis and pneumonia: results of a prospective, randomized, clinical trial.** *Crit Care Med* 1993, **21**:1132-1138.

55. Holzapfel L, Chastang C, Demingeon G, Bohe J, Piralla B, Coupry A: **A randomized study assessing the systematic search for maxillary sinusitis in nasotracheally mechanically ventilated patients. Influence of nosocomial maxillary sinusitis on the occurrence of ventilator-associated pneumonia.** *Am J Respir Crit Care Med* 1999, **159**:695-701.

56. Segers P, Speekenbrink RG, Ubbink DT, van Ogtrop ML, Bas A: **Prevention of nosocomial infection in cardiac surgery by decontamination of the nasopharynx and oropharynx with chlorhexidine gluconate.** *JAMA* 2006, **296**:2460-2466.

57. Seguin P, Tanguy M , Laviolle B, Tirel O, Malledant Y: **Effect of oropharyngeal decontamination by povidone-iodine on ventilator-associated pneumonia in patients with head trauma.** *Crit Care Med* 2006, **34**:1514-1519.

58. Valencia M, Ferrer M, Farre R, Navajas D, Badia JR, Nicolas JM, Torres A: **Automatic control of tracheal tube cuff pressure in ventilated patients in semirecumbent position: A randomized trial.** *Crit Care Med* 2007, **35**:1543-1549.

59. Brun-Buisson C, Legrand P, Rauss A, Richard C, Montravers F, Besbes M, Meakins JL, Soussy CJ, Lemaire F: **Intestinal decontamination for control of nosocomial multiresistant gram-negative bacilli. Study of an outbreak in an intensive care unit.** *Ann Intern Med* 1989, **110**:873-881.

60. de Smet AMGA, Kluytmans JAJW, Cooper BS, Mascini EM, Benus RFJ, van der Werf TS, van der Hoeven JG, Pickkers P, Bogaers-Hofman D, van der Meer NJ, Bernards AT, Kuijper EJ, Joore JC, Leverstein-van Hall MA, Bindels AJ, Jansz AR, Wesselink RM, de Jongh BM, Dennesen PJ, van Asselt GJ, te Velde LF, Frenay IH, Kaasjager K, Bosch FH, van Iterson M, Thijsen SF, Kluge GH, Pauw W, de Vries JW, Kaan JA, Arends JP, Aarts LP, Sturm PD, Harinck HI, Voss A, Uijtendaal EV, Blok HE, Thieme Groen ES, Pouw ME, Kalkman CJ, Bonten MJ: **Decontamination of the digestive tract and oropharynx in ICU patients.** *N Engl J Med* 2009, **360**:20–31.

61. Godard J, Guillaume C, Reverdy ME, Bachmann P, Bui-Xuan B, Nageotte A, Motin J: **Intestinal decontamination in a polyvalent ICU. A double-blind study.** *Intensive Care Med* 1990, **16**:307-311.

62. Nardi G, Di Silvestre A, De Monte A, Massarutti D, Proietti A, Troncon MG, Zussino M: **Reduction in gram-positive pneumonia and antibiotic consumption following the use of a SDD protocol including nasal and oral mupirocin.** *Eur J Emerg Med* 2001, **8**:203-214.

63. Oostdijk EA, Kesecioglu J, Schultz MJ, Visser CE, de Jonge E, van Essen EH, Bernards AT, Purmer I, Brimicombe R, Bergmans D, van Tiel F, Bosch FH, Mascini E, van Griethuysen A, Bindels A, Jansz A, van Steveninck FAL, van der Zwet WC, Fijen JW, Thijsen S, de Jong R, Oudbier J, Raben A, van der Vorm E, Koeman M, Rothbarth P, Rijkeboer A, Gruteke P, Hart-Sweet H, Peerbooms P, Winsser LJ, van Elsacker-Niele AMW, Demmendaal K, Brandenburg A, de Smet AMGA, Bonten MJM: **Effects of decontamination of the oropharynx and intestinal tract on antibiotic resistance in ICUs: a randomized clinical trial.** *JAMA* 2014, **312**:1429-1437.

64. Cerdá E, Abella A, Miguel A, Lorente JA, García-Hierro P, van Saene HK, Alia A, Aranguren A: **Enteral vancomycin controls methicillin-resistant Staphylococcus aureus endemicity in an intensive care burn unit: a 9-year prospective study.** *Ann Surg* 2007, **245**:397-407.

65. Fox MA, Peterson S, Fabri BM, Van Saene HK, Williets T: **Selective decontamination of the digestive tract in cardiac surgical patients.** *Crit Care Med* 1991, **19**:1486-90.

66. Hammond JM, Potgieter PD: **Long-term effects of selective decontamination on antimicrobial resistance.** *Crit Care Med* 1995, **23**:637-45.

67. Ledingham I, Eastaway A, Mckay I, Alcock S, Mcdonald J, Ramsay G: **Triple regimen of selective decontamination of the digestive tract, systemic cefotaxime, and microbiological surveillance for prevention of acquired infection in intensive care.** *Lancet* 1988, **1**:785-90.

68. McClelland P, Murray AE, Williams PS, van Saene HK, Gilbertson AA, Mostafa SM, Bone JM: **Reducing sepsis in severe combined acute renal and respiratory failure by selective decontamination of the digestive tract.** *Crit Care Med* 1990, **18**:935-9.

69. Silvestri L, Bragadin CM, Milanese M, Gregori D, Consales C, Gullo A, van Saene HFK: **Are most ICU infections really nosocomial? A prospective observational cohort study in mechanically ventilated patients.** *J Hosp Infect* 1999, **42**:125-133.

70. Silvestri L, Van Saene HKF, Milanese M, Fontana F, Gregori D, Oblach L, Blazic M: **Prevention of MRSA pneumonia by oral vancomycin decontamination: a randomised trial.** *Eur Resp J* 2004, **23**:921-926.

71. Stoutenbeek CP, van Saene HK, Miranda DR, Zandstra DF, Langrehr D: **The effect of oropharyngeal decontamination using topical nonabsorbable antibiotics on the incidence of nosocomial respiratory tract infections in multiple trauma patients.** *J Trauma* 1987, **27**:357-364.

72. Sydow M, Burchardi H, Crozier TA: **[The effect of selective decontamination on nosocomial infections, their causative agents and antibiotic resistance in long-term intubated intensive care patients].** *Anasth Intensivther Notfall Med* 1990, **25**:416-23.

73. van Patot HT, Leusink JA, Roodenburg J, De Jongh BM, Lau HS, de Boer S, De Boer A: **Selective decontamination of the digestive tract: effect of cessation of routine application at an ICU.** *Pharm World Sci* 1996, **18**:171-177.

74. Viviani M, Van Saene HK, Dezzoni R, Silvestri L, Di Lenarda R, Berlot G, Gullo A: **Control of imported and acquired methicillin-resistant Staphylococcus aureus (MRSA) in mechanically ventilated patients: a dose-response study of enteral vancomycin to reduce absolute carriage and infection.** *Anaesthesia and intensive care* 2005, **33**:361-372.

75. Barret JP, Jeschke MG, Herndon DN: **Selective decontamination of the digestive tract in severely burned pediatric patients.** *Burns* 2001, **27**:439-445.

76. Garbino J, Lew DP, Romand JA, Hugonnet S, Auckenthaler R, Pittet D: **Prevention of severe Candida infections in nonneutropenic, high-risk, critically ill patients: a randomized, double-blind, placebo-controlled trial in patients treated by selective digestive decontamination.** *Intensive Care Med* 2002, **28**:1708-1717.

77. Gaussorgues P, Salord M, Sirodot S, Tigaud S, Cagnin S, Gerard M, Robert D: **Efficiency of selective decontamination of the digestive tract on the occurrence of nosocomial bacteremia in patients on mechanical ventilation receiving betamimetic therapy.** *Réan Soins Intens Méd Urg* 1991, **7**:169-174.

78. Korinek AM, Laisne MJ, Nicolas MH, Raskine L, Deroin V, Sanson-Lepors MJ: **Selective decontamination of the digestive tract in neurosurgical intensive care unit patients: a double-blind, randomized, placebo-controlled study.** *Crit Care Med* 1993, **21**:1466-1473.

79. Langlois-Karaga A, Bues-Charbit M, Davignon A, Albanese J, Durbec O, Martin C, Balansard G: **Selective digestive decontamination in multiple trauma patients: cost and efficacy.** *Pharm World Sci* 1995, **17**:12-16.

80. Quinio B, Albanese J, Bues-Charbit M, Viviand X, Martin C: **Selective decontamination of the digestive tract in multiple trauma patients. A prospective double-blind, randomized, placebo-controlled study.** *Chest* 1996, **109**:765-772.

81. Rodriguez-Roldan JM, Altuna-Cuesta A, Lopez A, Carrillo A, Garcia J, Leon J, Martinez-Pellus AJ: **Prevention of nosocomial lung infection in ventilated patients: use of an antimicrobial pharyngeal nonabsorbable paste.** *Crit Care Med* 1990, **18**:1239-1242.

82. Ruza F, Alvarado F, Herruzo R, Delgado MA, Garcia S, Dorao P, Goded F: **Prevention of nosocomial infection in a pediatric intensive care unit (PICU) through the use of selective digestive decontamination.** *Eur J Epidemiol* 1998, **14**:719-27.

83. Wiener J, Itokazu G, Nathan C, Kabins SA, Weinstein RA: **A randomized, double-blind, placebo-controlled trial of selective digestive decontamination in a medical-surgical intensive care unit**. *Clin Infect Dis* 1995, **20**:861-867.

84. Aerdts SJ, van Dalen R, Clasener HA, Festen J, van Lier HJ, Vollaard EJ: **Antibiotic prophylaxis of respiratory tract infection in mechanically ventilated patients. A prospective, blinded, randomized trial of the effect of a novel regimen.** *Chest* 1991, **100**:783-791.

85. Blair P, Rowlands BJ, Lowry K, Webb H, Armstrong P, Smilie J: **Selective decontamination of the digestive tract: a stratified, randomized, prospective study in a mixed intensive care unit.** *Surgery* 1991, **110**:303-309.

86. Cerra FB, Maddaus MA, Dunn DL, Wells CL, Konstantinides NN, Lehmann SL, Mann HJ: **Selective gut decontamination reduces nosocomial infections and length of stay but not mortality or organ failure in surgical intensive care unit patients.** *Arch Surg* 1992, **127**:163-167.

87. Cockerill FR, 3rd, Muller SR, Anhalt JP, Marsh HM, Farnell MB, Mucha P, Gillespie DJ, Ilstrup DM, Larson-Keller JJ, Thompson RL: **Prevention of infection in critically ill patients by selective decontamination of the digestive tract.** *Ann Intern Med* 1992, **117**:545-553.

88. de La Cal MA, Cerda E, Garcia-Hierro P, Van Saene HK, Gómez-Santos D, Negro E, Lorente JA: **Survival benefit in critically ill burned patients receiving selective decontamination of the digestive tract: a randomized, placebo-controlled, double-blind trial.** *Ann Surg* 2005, **241**:424-430.

89. Finch RG, Tomlinson P, Holliday M, Sole K, Stack C, Rocker G: **Selective decontamination of the digestive tract (SDD) in the prevention of secondary sepsis in a medical/surgical intensive care unit** [abstract]. Seventeenth international congress of chemotherapy, Berlin, 1991:No 0471.

90. Flaherty J, Nathan C, Kabins SA, Weinstein RA: **Pilot trial of selective decontamination for prevention of bacterial infection in an intensive care unit.** *J Infect Dis* 1990, **162**:1393-1397.

91. Jacobs S, Foweraker JE, Roberts SE: **Effectiveness of selective decontamination of the digestive tract (SDD) in an ICU with a policy encouraging a low gastric pH.** *Clin Intensive Med* 1992, **3**:52-58.

92. Kerver AJ, Rommes JH, Mevissen-Verhage EA, Hulstaert PF, Vos A, Verhoef J, Wittebol P: **Prevention of colonization and infection in critically ill patients: a prospective randomized study.** *Crit Care Med* 1988, **16**:1087-1093.

93. Krueger WA, Lenhart FP, Neeser G, Ruckdeschel G, Schreckhase H, Eissner HJ, Forst H, Eckart J, Peter K, Unertl KE**: Influence of combined intravenous and topical antibiotic prophylaxis on the incidence of infections, organ dysfunctions, and mortality in critically ill surgical patients: a prospective, stratified, randomized, double-blind, placebo-controlled clinical trial.** *Am J Respir Crit Care Med* 2002, **166**:1029-1037.

94. Palomar M, Alvarez-Lerma F, Jorda R, Bermejo B, for the Catalan Study Group of Nosocomial Pneumonia Prevention: **Prevention of nosocomial infection in mechanically ventilated patients: selective digestive decontamination versus sucralfate.** *Clin Intensive Care* 1997, **8**:228-235.

95. Rocha LA, Martin MJ, Pita S, Paz J, Seco C, Margusino L, Villanueva R, Duran MT: **Prevention of nosocomial infection in critically ill patients by selective decontamination of the digestive tract. A randomized, double blind, placebo-controlled study.** *Intensive Care Med* 1992, **18**:398-404.

96. Rolando N, Gimson A, Wade J, Philpott - Howard J, Casewell M, Williams R: **Prospective controlled trial of selective parenteral and enteral antimicrobial regimen in fulminant liver failure.** *Hepatol* 1993, **17**:196-201.

97. Sanchez Garcia M, Cambronero Galache JA, Lopez Diaz J, Cerda Cerda E, Rubio Blasco J, Gomez Aguinaga MA, Nunez Reiz A, Rogero Marin S, Onoro Canaveral JJ, Sacristan del Castillo JA: **Effectiveness and cost of selective decontamination of the digestive tract in critically ill intubated patients. A randomized, double-blind, placebo-controlled, multicenter trial.** *Am J Respir Crit Care Med* 1998, **158**:908-916.

98. Stoutenbeek CP, van Saene HKF, Little RA, Whitehead A: **The effect of selective decontamination of the digestive tract on mortality in multiple trauma patients: a multicenter randomized controlled trial.** *Intensive Care Med* 2007, **33**;261-70.

99. Ulrich C, Harinck-de Weerd JE, Bakker NC, Jacz K, Doornbos L, de Ridder VA: **Selective decontamination of the digestive tract with norfloxacin in the prevention of ICU-acquired infections: a prospective randomized study.** *Intensive Care Med* 1989, **15**:424-431.

100. Verwaest C, Verhaegen J, Ferdinande P, Schetz M, Van den Berghe G, Verbist L, Lauwers P: **Randomized, controlled trial of selective digestive decontamination in 600 mechanically ventilated patients in a multidisciplinary intensive care unit.** *Crit Care Med* 1997, **25**:63-71.

101. Zobel G, Kuttnig M, Grubbauer HM, Semmelrock HJ, Thiel W: **Reduction of colonization and infection rate during pediatric intensive care by selective decontamination of the digestive tract.** *Crit Care Med* 1991, **19**:1242-1246.

102. Arnow PM, Carandang GC, Zabner R, Irwin ME: **Randomized controlled trial of selective bowel decontamination for prevention of infections following liver transplantation.** *Clin Infect Dis* 1996, **22**:997-1003.

103. Bion JF, Badger I, Crosby HA, Hutchings P, Kong KL, Baker J, Hutton P, McMaster P, Buckels JA, Elliott TSJ: **Selective decontamination of the digestive tract reduces gram-negative pulmonary colonization but not systemic endotoxemia in patients undergoing elective liver transplantation.** *Crit Care Med* 1994, **22**:40-9.

104. Ferrer M, Torres A, Gonzalez J, Puig de la Bellacasa J, el-Ebiary M, Roca M, Gatell JM, Rodriguez-Roisin R: **Utility of selective digestive decontamination in mechanically ventilated patients.** *Ann Intern Med* 1994, **120**:389-395.

105. Georges B, Mazerolles M, Decun J-F, Rouge P, Pomies S, Cougot P: **Décontamination digestive sélective résultats d'une étude chez le polytraumatisé.** *Réanimation Soins Intensifs Médecin d'Urgence* 1994, **3**:621-627.

106. Hammond JM, Potgieter PD, Saunders GL, Forder AA: **Double-blind study of selective decontamination of the digestive tract in intensive care.** *Lancet* 1992, **340**:5-9.

107. Hartenauer U, Thulig B, Diemer W, Lawin P, Fegeler W, Kehrel R, Ritzerfeld W: **Effect of selective flora suppression on colonization, infection, and mortality in critically ill patients: a one-year, prospective consecutive study.** *Crit Care Med* 1991, **19**:463-73.

108. Hellinger WC, Yao JD, Alvarez S, Blair JE, Cawley JJ, Paya CV, O'Brien PC: **A randomized, prospective, double-blinded evaluation of selective bowel decontamination in liver transplantation.** *Transplantation* 2002, **73**:1904-9.

109. Laggner AN, Tryba M, Georgopoulos A, Lenz K, Grimm G, Graninger W, Schneeweiss B, Druml W: **Oropharyngeal decontamination with gentamicin for long-term ventilated patients on stress ulcer prophylaxis with sucralfate?** *Wien Klin Wochenschr* 1994, **106**:15-19.

110. Rolando N, Wade JJ, Stangou A, Gimson AE, Wendon J, Philpott - Howard J, Williams R: **Prospective study comparing the efficacy of prophylactic parenteral antimicrobials, with or without enteral decontamination, in patients with acute liver failure.** *Liver transpl Surg* 1996, **2**:8-13.

111. Stoutenbeek CP, van Saene HKF, Zandstra DF: **Prevention of multiple organ failure by selective decontamination of the digestive tract in multiple trauma patients.** In The Immune Consequences of Shock, Trauma and Sepsis. Volume 2. Edited by Faist E, Baue AE, Schildberg FW. Lengerich: Pabst Science; 1996:1055-66.

112. Tetteroo GWM, Castelein A, Tilanus HW, Ince C, Bruining HA, Wagenvoort JHT: **Selective decontamination to reduce gram-negative colonisation and infections after oesophageal resection.** *Lancet* 1990, **335**:704-7.

113. Verhaegen J. **Randomized study of selective digestive decontamination on colonization and prevention of infections in mechanically ventilated patients in the ICU.** Doctor of Medical Science thesis. University hospital, Leuven 1992.
