# Supplementary material for: Topical antibiotics as a major contextual hazard toward bacteremia within selective digestive decontamination studies: a meta-analysis
Source: BMC Infect Dis. 2014 Dec 31;14:714. doi: 10.1186/s12879-014-0714-x (PMC4300056; doi:10.1186/s12879-014-0714-x)
Supplement: Supplementary file 2 — Additional file 2: Caterpillar plots for VAP-IP data (Figures S1-4). (PDF 26 KB) [file 12879_2014_714_MOESM2_ESM.pdf]

Additional file 2: Table of contents

Figure S1: VAP-incidence proportion: observational studies (Benchmark groups) 2

Figure S2: VAP-incidence proportion: studies of VAP prevention using non-antibiotic methods 3

Figure S3: VAP-incidence proportion: control groups of studies of VAP prevention using SDD 4

Figure S4: VAP-incidence proportion: intervention groups of studies of VAP prevention using SDD 5

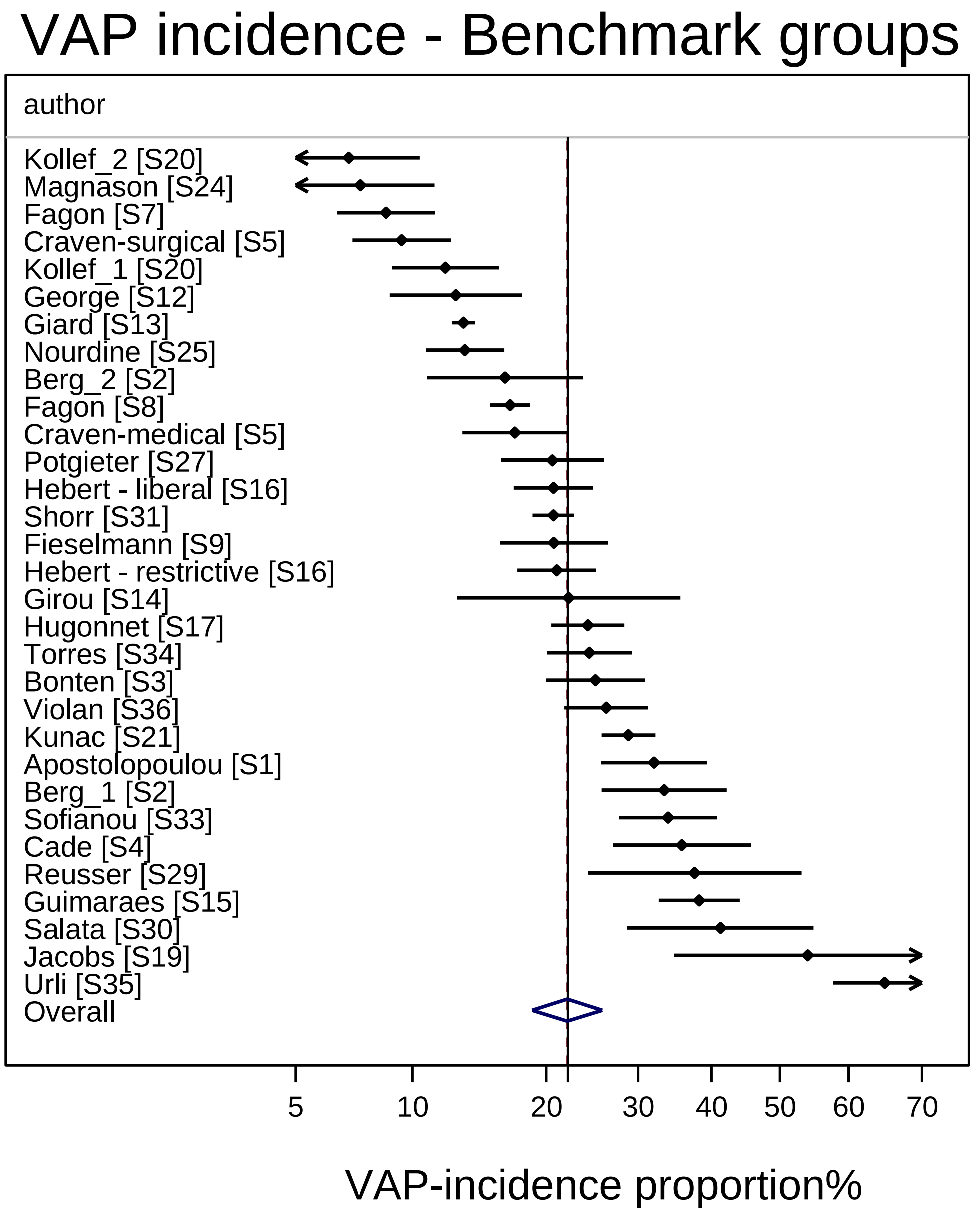

Figure S1. Caterpillar plots of the group specific (small diamonds) and summary (large open diamond, broken vertical line) VAP-incidence proportion and 95 % CI of observational studies (Benchmark groups). Studies are listed in Table S1. Note that the x axis is a logit scale.

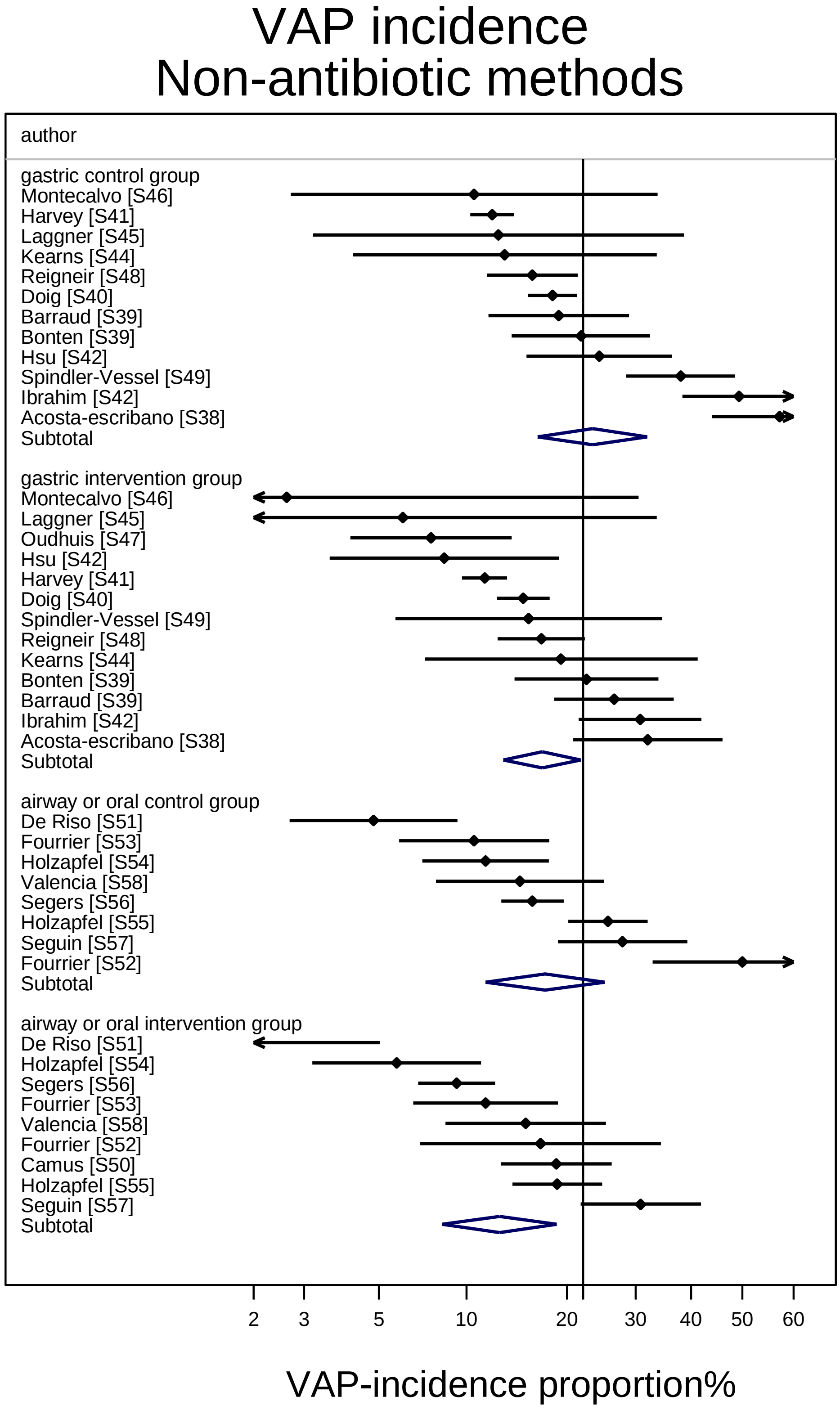

Figure S2. Caterpillar plots of the group specific (small diamonds) and summary (large open diamond, broken vertical line) VAP-incidence proportion and 95 % CI of control and intervention groups of studies of VAP prevention using non-antibiotic methods. For comparison, the summary VAP -IP (vertical line) derived from the benchmark groups from figure S1 is shown. Studies are listed in Table S2. Note that the x axis is a logit scale.

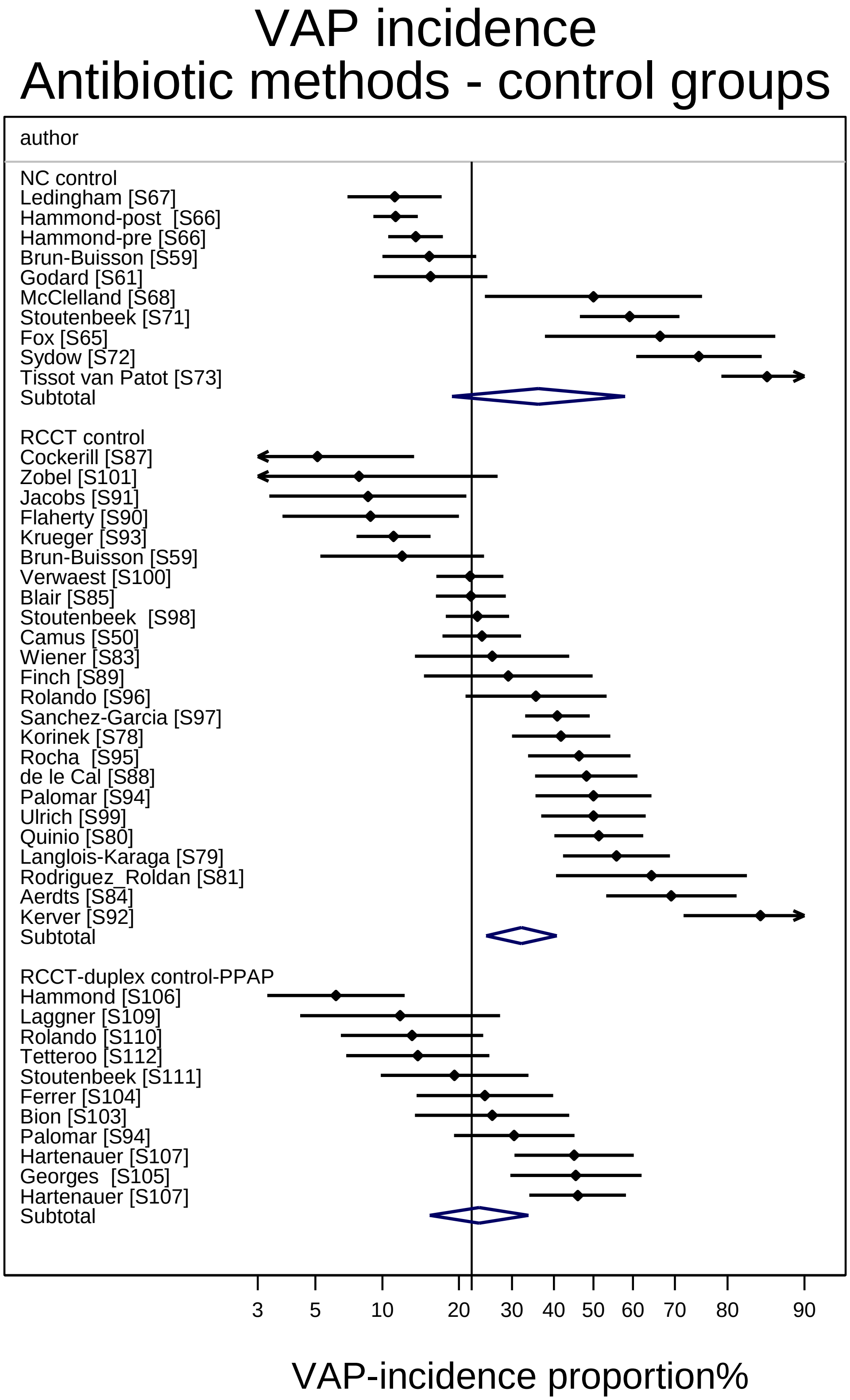

Figure S3. Caterpillar plots of the group specific (small diamonds) and summary (large open diamond) VAP-incidence proportion and 95 % CI of control groups of studies of VAP prevention using SDD for which the control groups did not or did receive topical placebo. For comparison, the summary VAP- IP (vertical line) derived from the benchmark groups from figure S1 are shown. Studies are listed in Tables S3, S4 & S5. Note that the x axis is a logit scale.

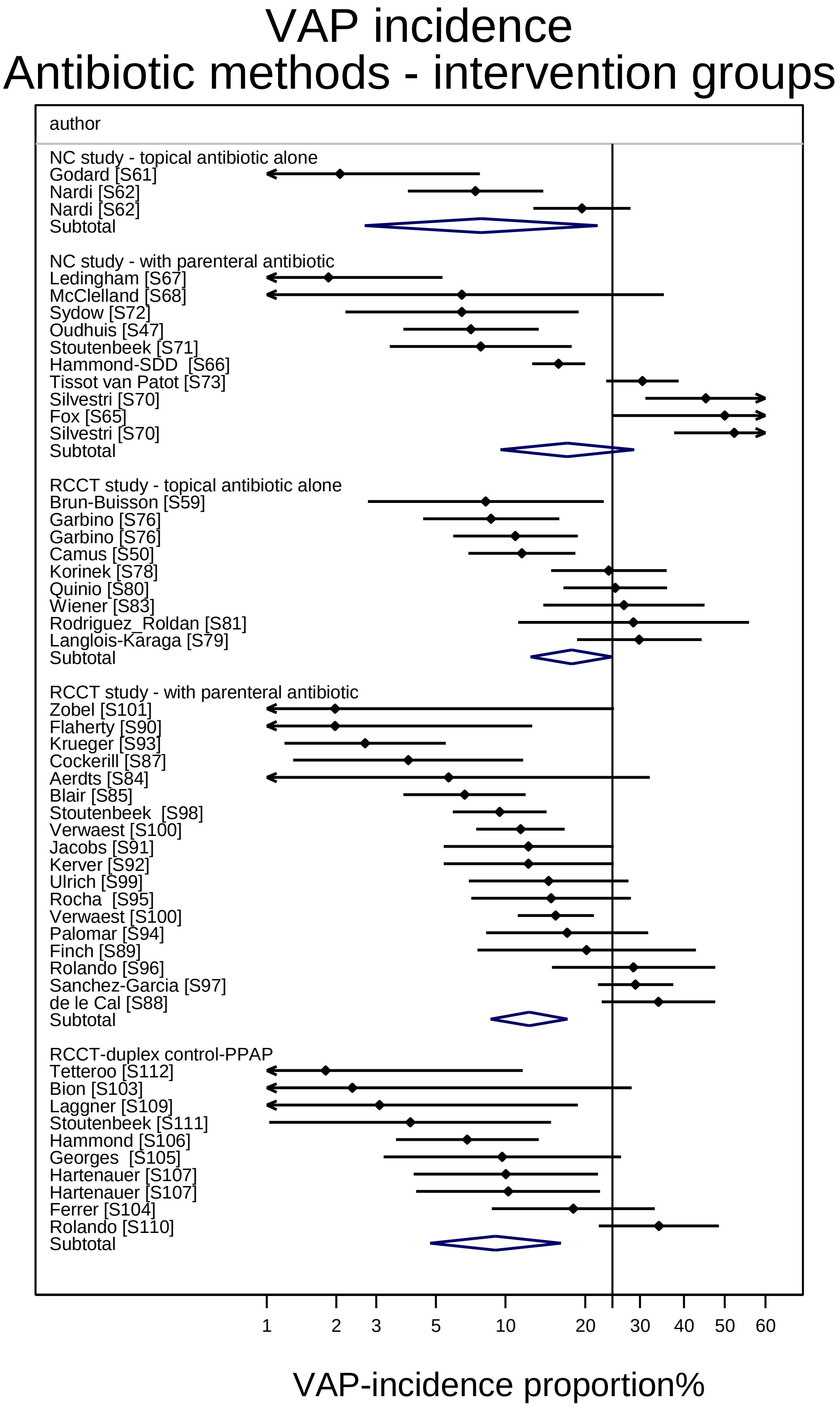

Figure S4. Caterpillar plots of the group specific (small diamonds) and summary (large open diamond) VAP-incidence proportion and 95 % CI of intervention groups of studies of VAP prevention using SDD for which the control groups did not or did receive topical placebo. For comparison, the summary VAP - IP (vertical line) derived from the benchmark groups from figure S1 are shown. Studies are listed in Tables S3, S4 and S5. Note that the x axis is a logit scale.
