# Supplementary material for: Topical antibiotics as a major contextual hazard toward bacteremia within selective digestive decontamination studies: a meta-analysis
Source: BMC Infect Dis. 2014 Dec 31;14:714. doi: 10.1186/s12879-014-0714-x (PMC4300056; doi:10.1186/s12879-014-0714-x)
Supplement: Supplementary file 3 — Additional file 3: Forrest plots showing counterfactual effects as odds ratios between control versus intervention group VAP incidence (Figures S5, S7 & S9) and bacteremia incidence (Figures S6, S8 & S10) as study specific and summary effect sizes. (PDF 82 KB) [file 12879_2014_714_MOESM3_ESM.pdf]

Additional file 3: Table of contents

Figure S5. Effect size of non-antibiotic methods on VAP-incidence 2

Figure S6. Effect size of non-antibiotic methods on bacteremia-incidence 3

Figure S7. Effect size of SDD on VAP-incidence: non-concurrent design studies 4

Figure S8. Effect size of SDD on bacteremia-incidence: non-concurrent design studies 5

Figure S9. Effect size of SDD on VAP-incidence: RCCT’s 6

Figure S10. Effect size of SDD on bacteremia-incidence: RCCT’s 7

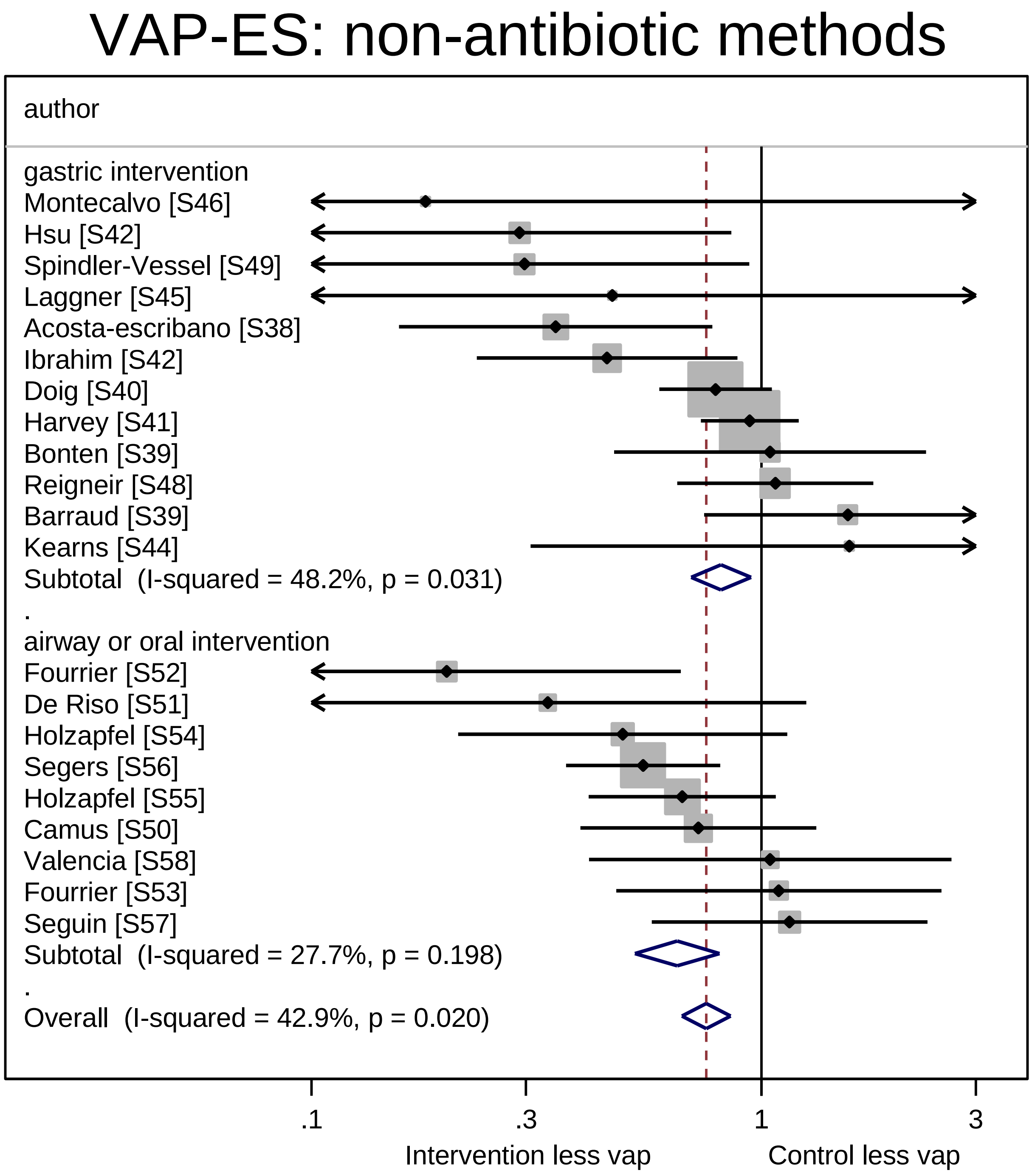

Figure S5. Effect size of non-antibiotic methods of VAP prevention on the incidence of VAP as odds ratios for studies as listed in Table S2. Square symbols are proportional to study size and associated whiskers are 95% confidence intervals. Study weighting used in the calculation of the summary odds ratio is based on the inverse of the study variance. The open diamonds represent the sub-group and overall summary odds ratios and associated 95% confidence intervals. The overall summary odds ratio is also represented by the vertical broken line.

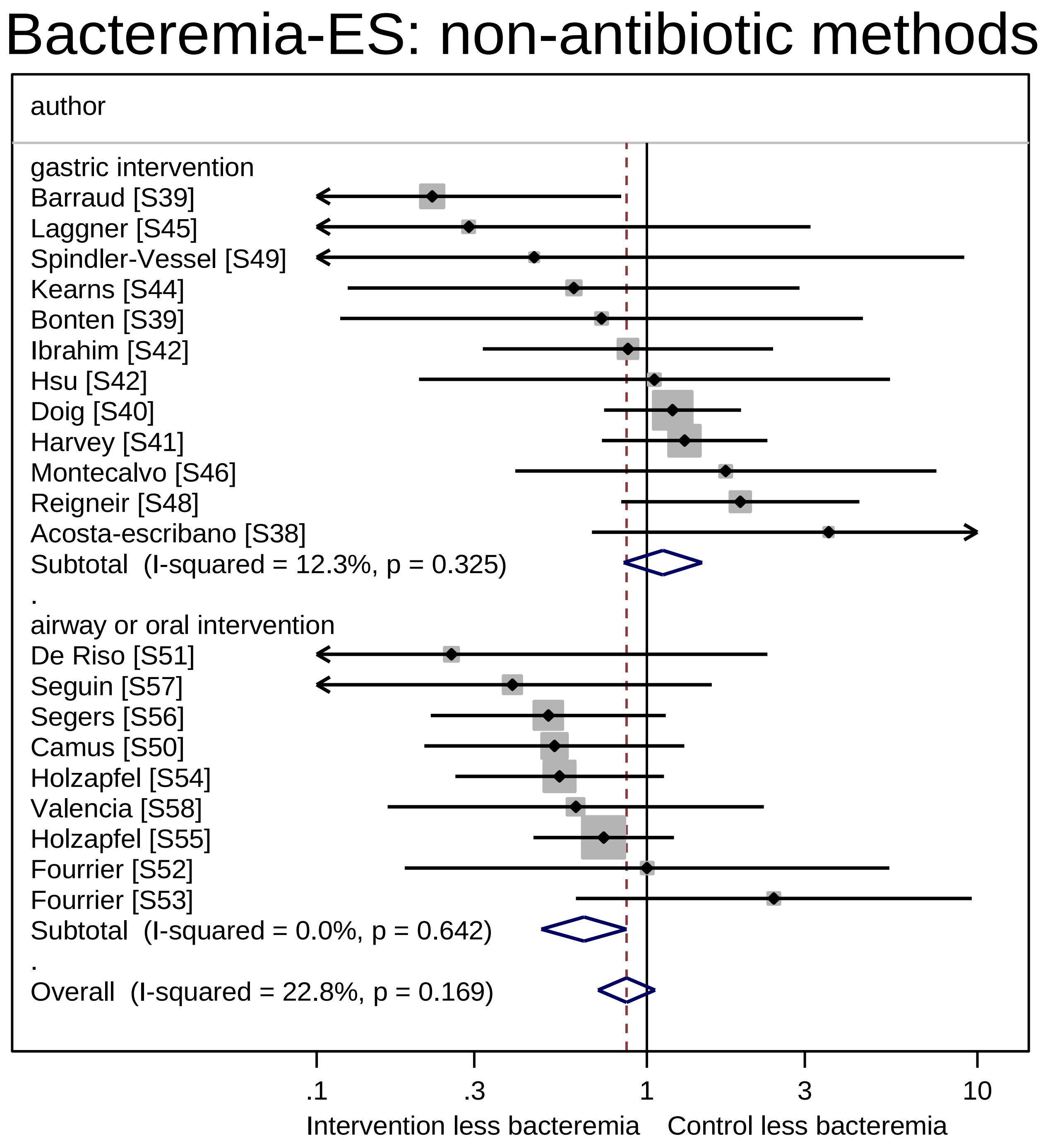

Figure S6. Effect size of non-antibiotic methods of VAP prevention on the incidence of bacteremia as odds ratios for studies as listed in Table S2. Details as for Figure S5.

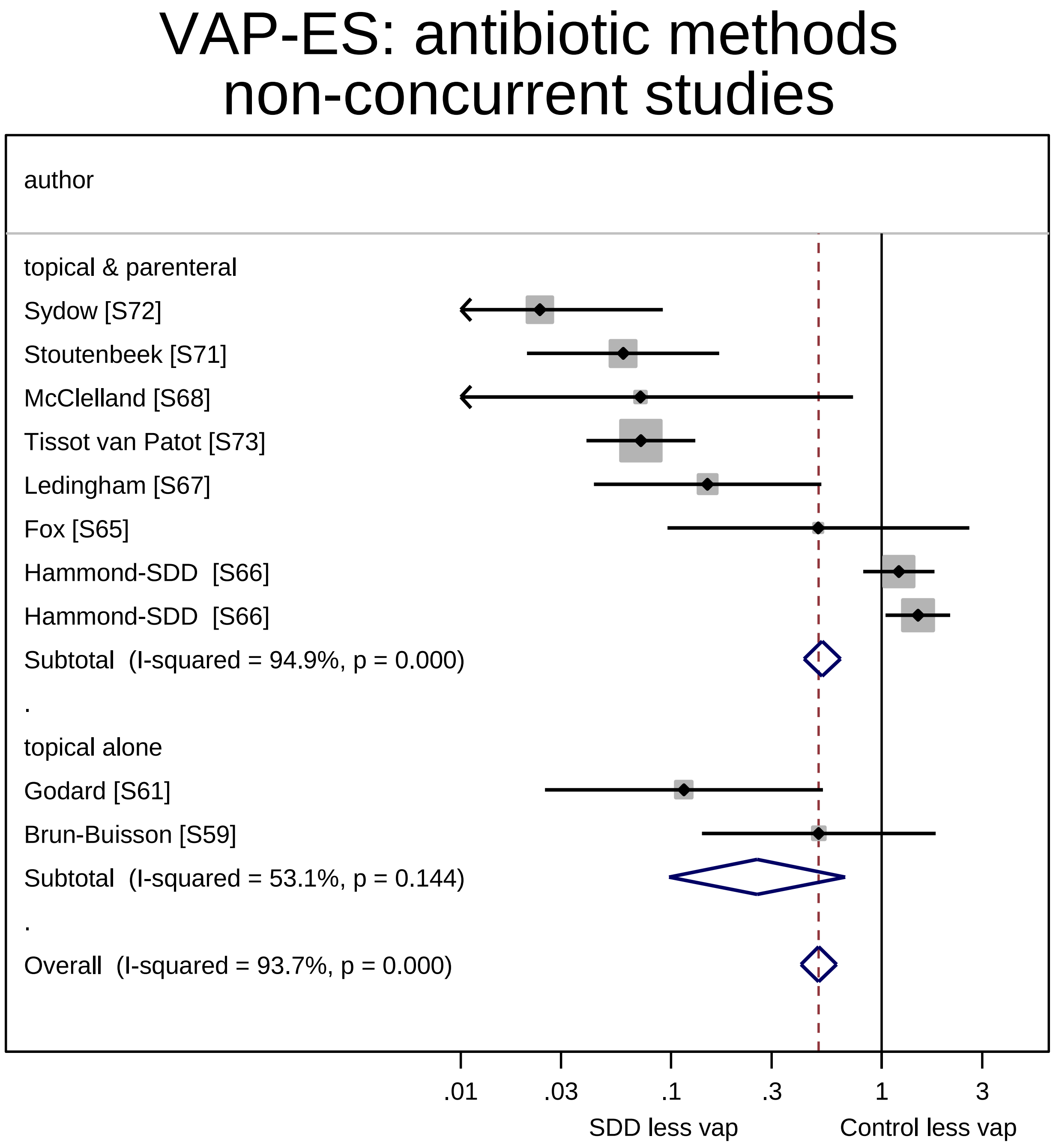

Figure S7. Effect size of antibiotic methods of VAP prevention using SDD on the incidence of VAP as odds ratios in studies with non-concurrent controls as listed in Table S3. Details as for Figure S5.

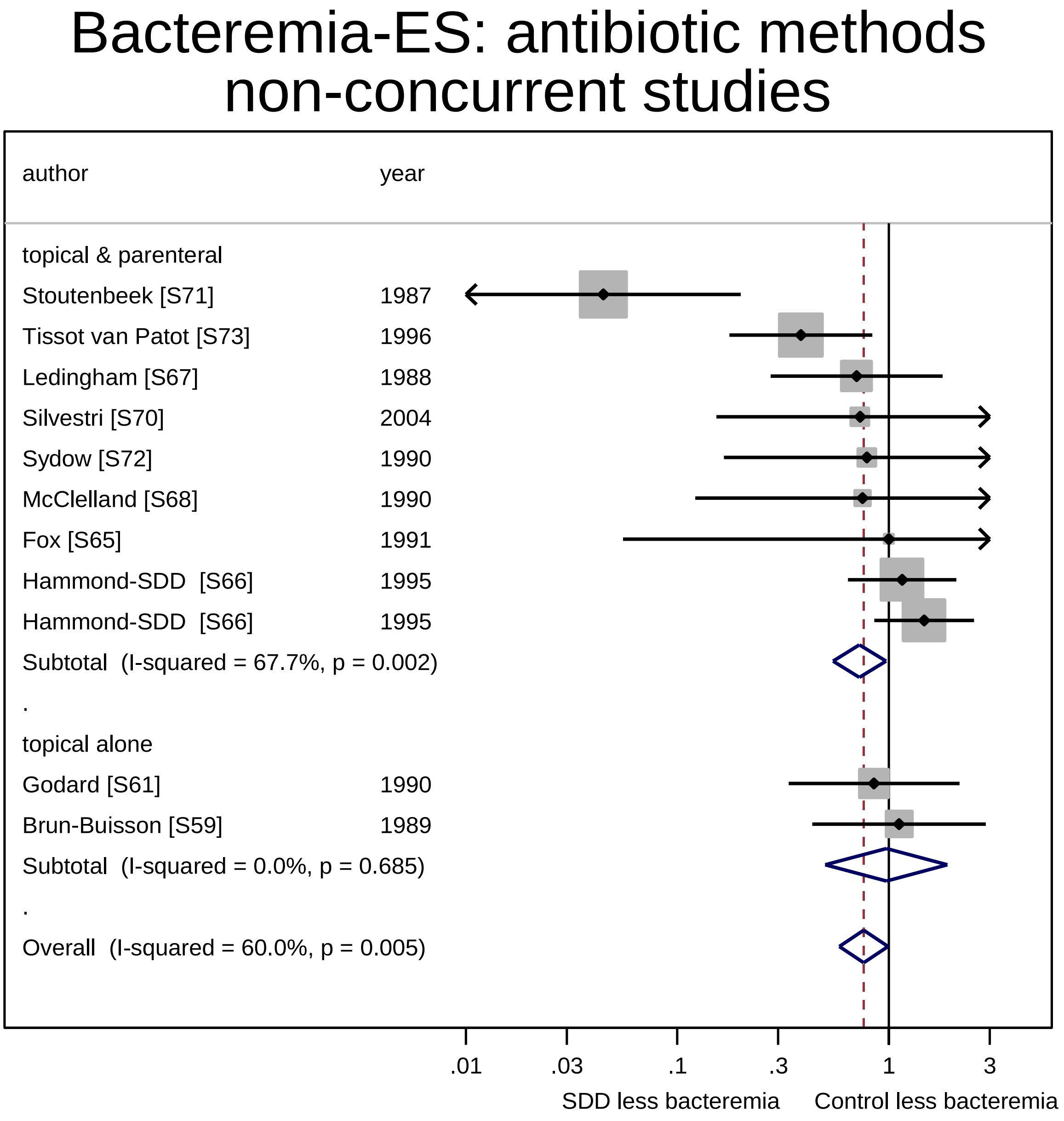

Figure S8. Effect size of antibiotic methods of VAP prevention using SDD on the incidence of bacteremia as odds ratios in studies with non-concurrent controls as listed in Table S3. Details as for Figure S5.

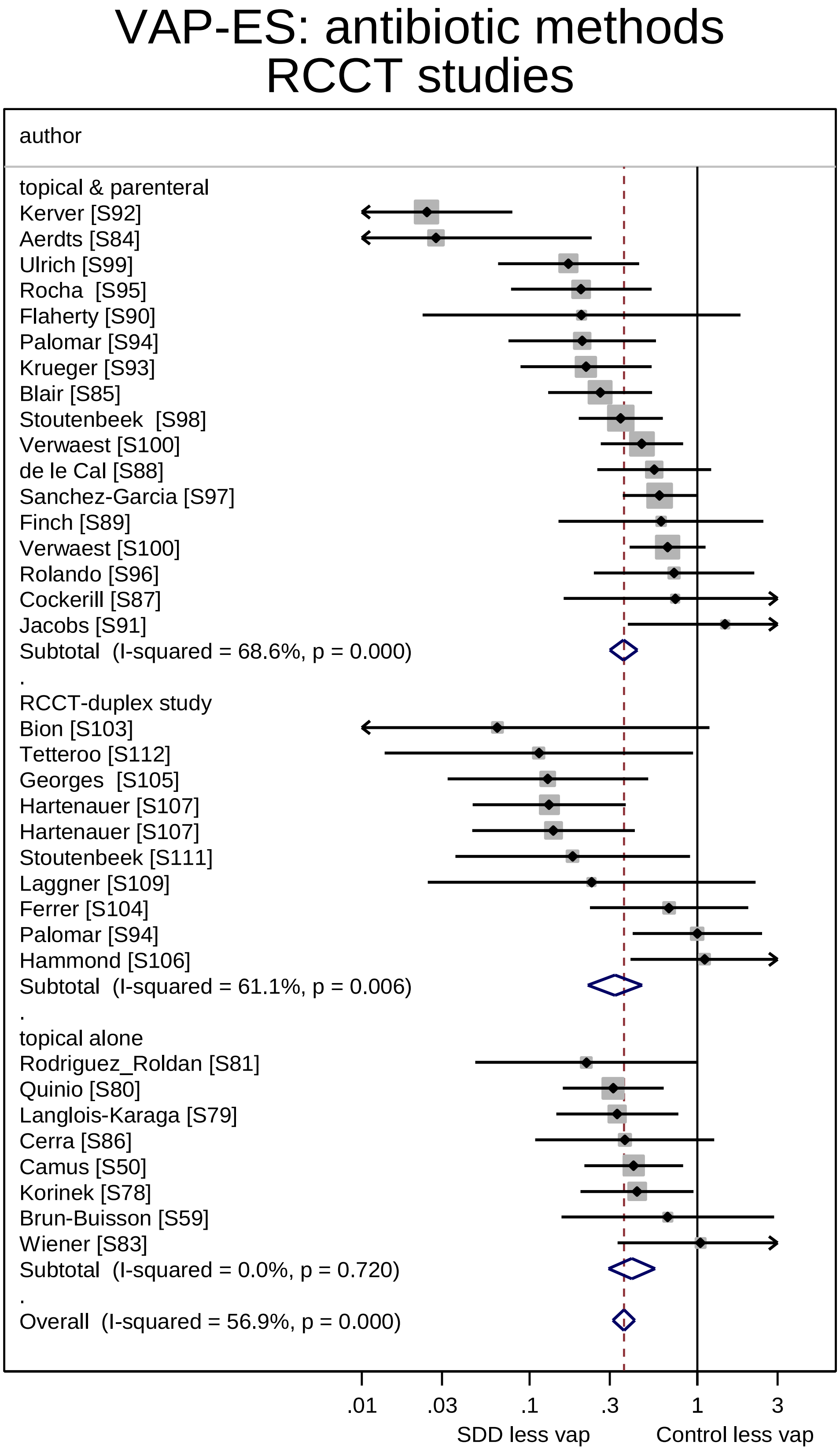

Figure S9. Effect size of antibiotic methods of VAP prevention using SDD on the incidence of VAP as odds ratios in studies with concurrent controls as listed in Tables S4 & S5.
Details as for Figure S5.

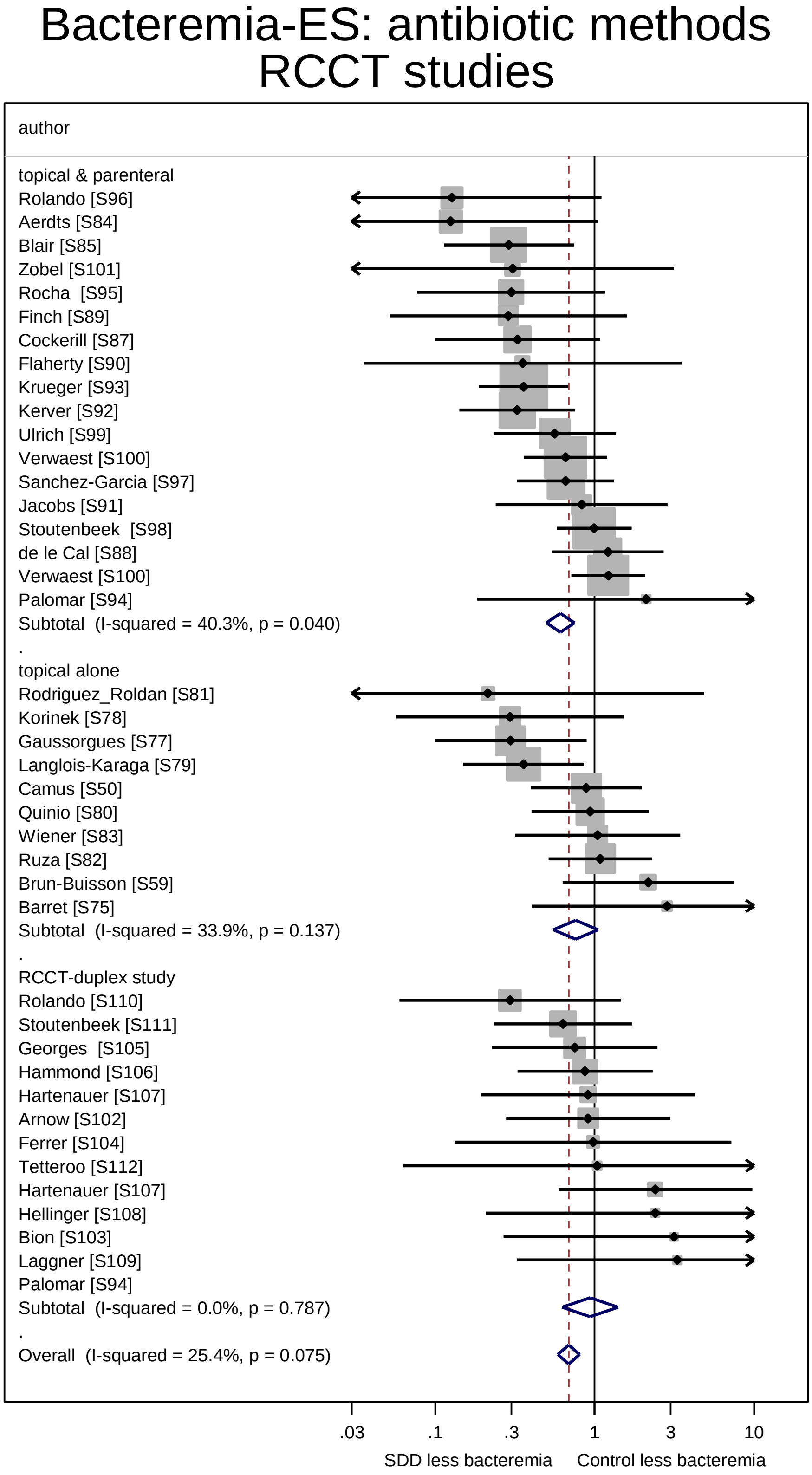

Figure S10. Effect size of antibiotic methods of VAP prevention using SDD on the incidence of bacteremia as odds ratios in studies with concurrent controls as listed in Tables S4 & S5. Details as for Figure S5.
