# Supplementary figures and images for: Topical antibiotics as a major contextual hazard toward bacteremia within selective digestive decontamination studies: a meta-analysis
Source: BMC Infect Dis. 2014 Dec 31;14:714. doi: 10.1186/s12879-014-0714-x (PMC4300056; doi:10.1186/s12879-014-0714-x)

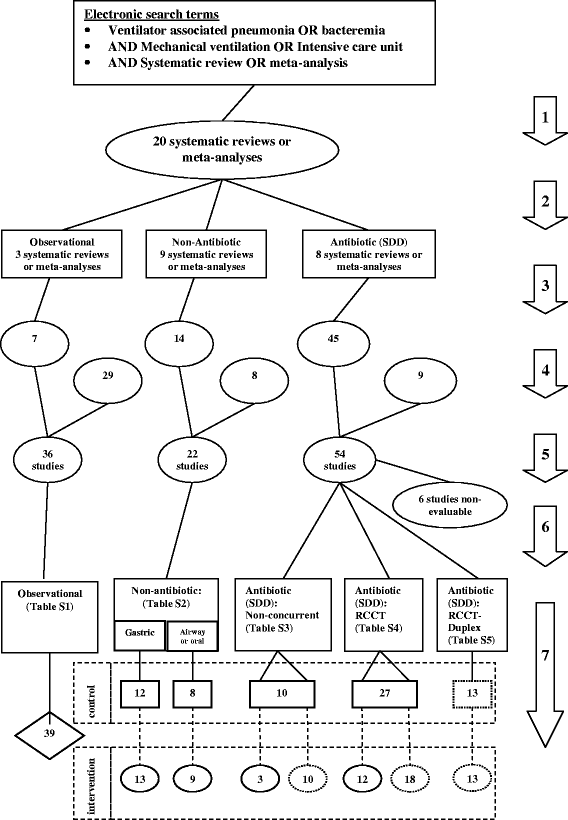

Supplement: Supplementary file 4 — Authors’ original file for figure 1 [file 12879_2014_714_MOESM4_ESM.gif]

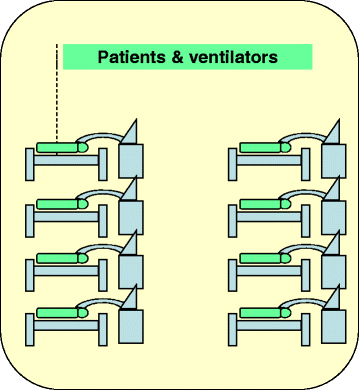

Supplement: Supplementary file 5 — Authors’ original file for figure 2 [file 12879_2014_714_MOESM5_ESM.gif]

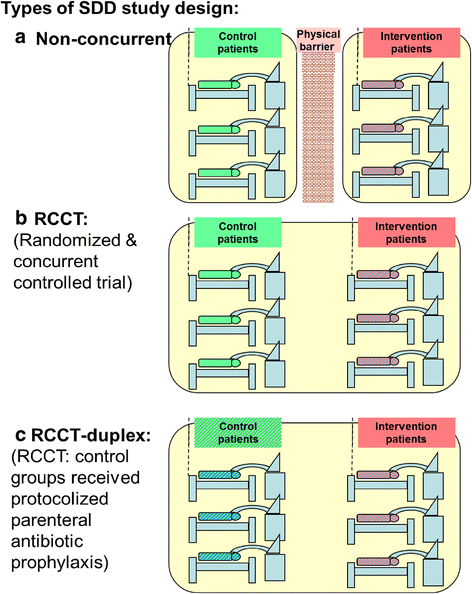

Supplement: Supplementary file 6 — Authors’ original file for figure 3 [file 12879_2014_714_MOESM6_ESM.gif]

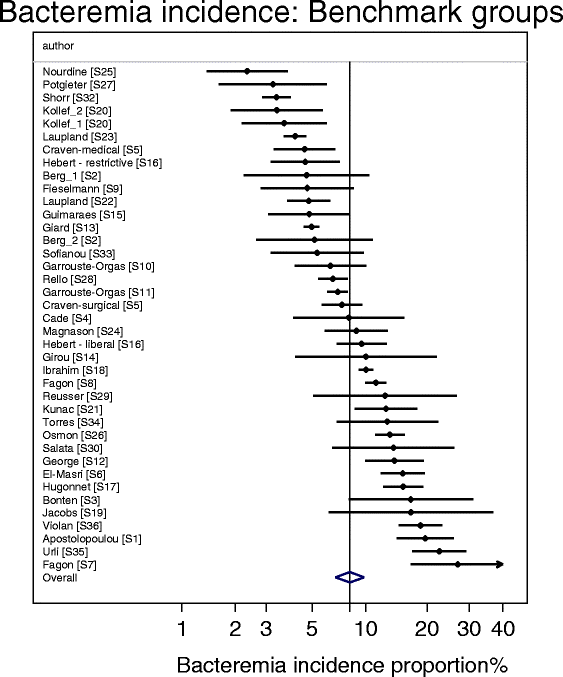

Supplement: Supplementary file 7 — Authors’ original file for figure 4 [file 12879_2014_714_MOESM7_ESM.gif]

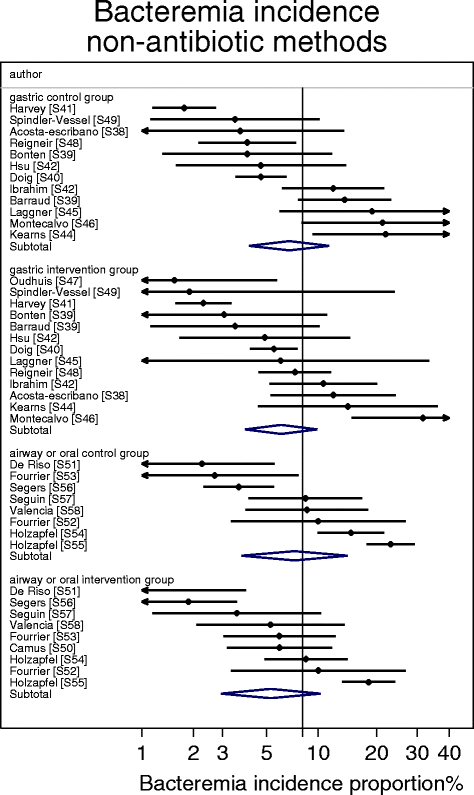

Supplement: Supplementary file 8 — Authors’ original file for figure 5 [file 12879_2014_714_MOESM8_ESM.gif]

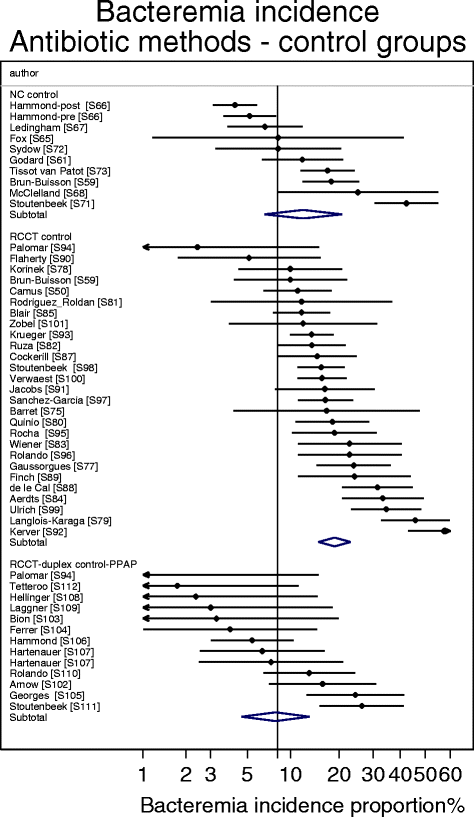

Supplement: Supplementary file 9 — Authors’ original file for figure 6 [file 12879_2014_714_MOESM9_ESM.gif]

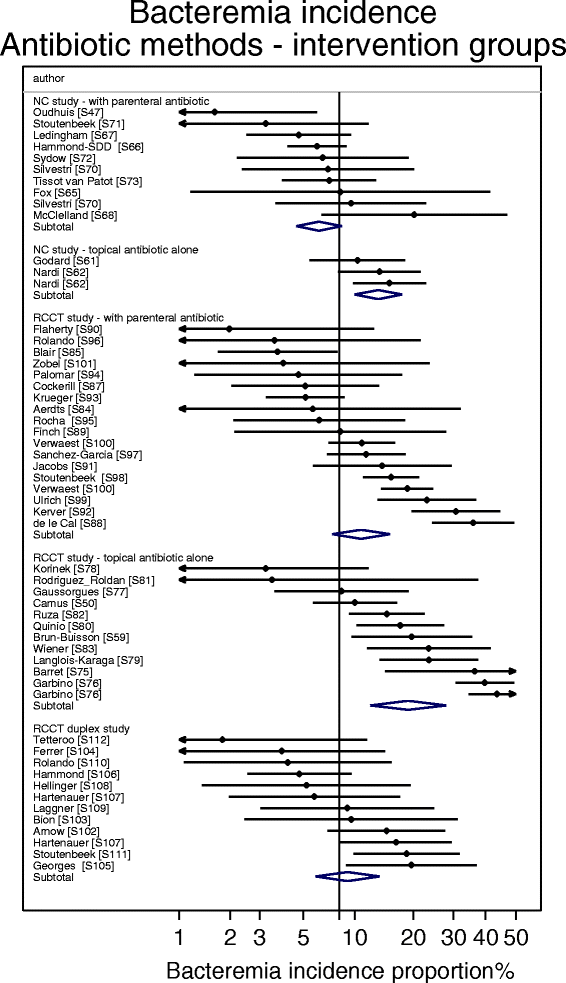

Supplement: Supplementary file 10 — Authors’ original file for figure 7 [file 12879_2014_714_MOESM10_ESM.gif]

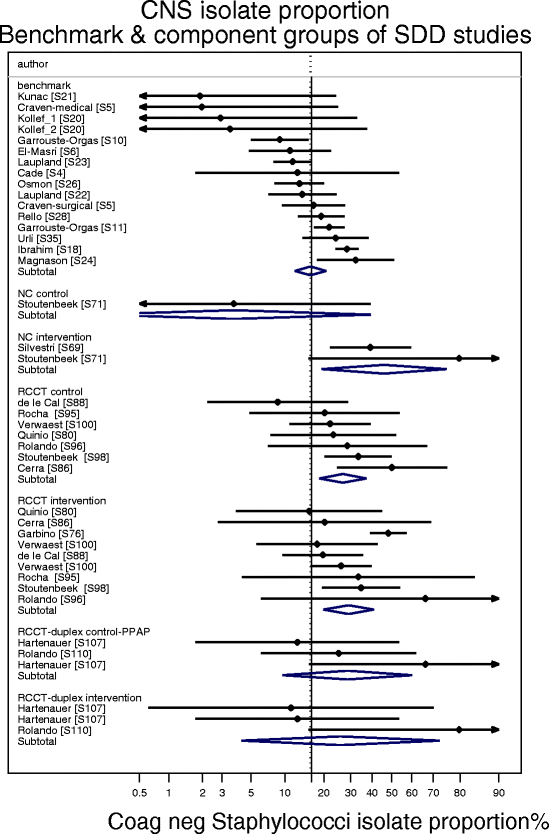

Supplement: Supplementary file 11 — Authors’ original file for figure 8 [file 12879_2014_714_MOESM11_ESM.gif]

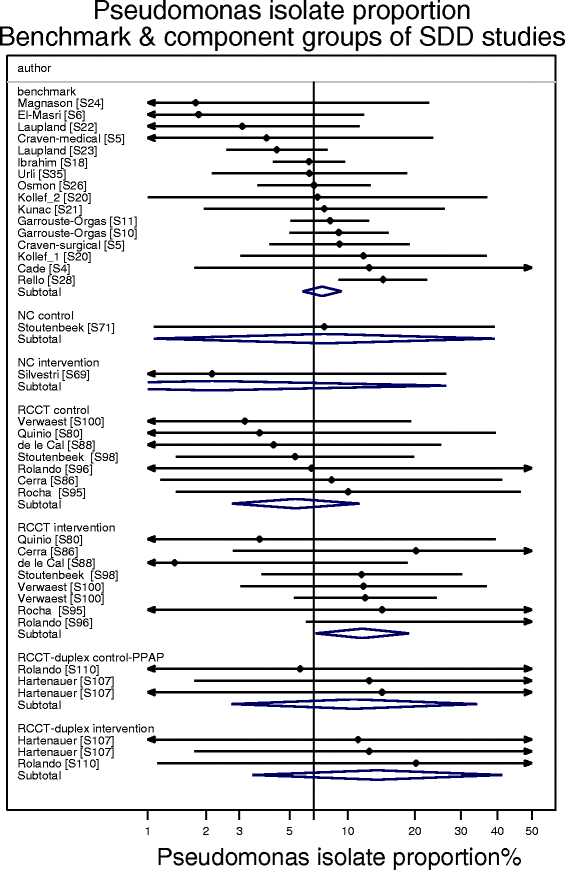

Supplement: Supplementary file 12 — Authors’ original file for figure 9 [file 12879_2014_714_MOESM12_ESM.gif]
